# Hypoxia drives transient site-specific copy gain and drug-resistant gene expression

**DOI:** 10.1101/gad.259796.115

**Published:** 2015-05-15

**Authors:** Joshua C. Black, Elnaz Atabakhsh, Jaegil Kim, Kelly M. Biette, Capucine Van Rechem, Brendon Ladd, Paul d. Burrowes, Carlos Donado, Hamid Mattoo, Benjamin P. Kleinstiver, Bing Song, Grasiella Andriani, J. Keith Joung, Othon Iliopoulos, Cristina Montagna, Shiv Pillai, Gad Getz, Johnathan R. Whetstine

**Affiliations:** 1 Massachusetts General Hospital Cancer Center, Department of Medicine, Harvard Medical School, Charlestown, Massachusetts 02129, USA;; 2 Broad Institute of Massachusetts Institute of Technology and Harvard, Cambridge, Massachusetts 02142, USA;; 3 Massachusetts General Hospital Cancer Center, Department of Pathology, Harvard Medical School, Charlestown, Massachusetts 02129, USA;; 4 Massachusetts General Hospital Center for Computational and Integrative Biology, Charlestown, Massachusetts 02129, USA;; 5 Department of Genetics, Pathology, Albert Einstein College of Medicine, Yeshiva University, Bronx, New York 10461, USA;; 6 Department of Medicine, Division of Hematology-Oncology, Massachusetts General Hospital, Boston, Massachusetts 02114, USA

**Keywords:** CNV, JmjC, KDM4A, hypoxia, site-specific copy gain, tumor heterogeneity

## Abstract

Copy number heterogeneity is a prominent feature within tumors. The molecular basis for this heterogeneity remains poorly characterized. Here, we demonstrate that hypoxia induces transient site-specific copy gains (TSSGs) in primary, nontransformed, and transformed human cells. Hypoxia-driven copy gains are not dependent on HIF1α or HIF2α; however, they are dependent on the KDM4A histone demethylase and are blocked by inhibition of KDM4A with a small molecule or the natural metabolite succinate. Furthermore, this response is conserved at a syntenic region in zebrafish cells. Regions with site-specific copy gain are also enriched for amplifications in hypoxic primary tumors. These tumors exhibited amplification and overexpression of the drug resistance gene *CKS1B*, which we recapitulated in hypoxic breast cancer cells. Our results demonstrate that hypoxia provides a biological stimulus to create transient site-specific copy alterations that could result in heterogeneity within tumors and cell populations. These findings have major implications in our understanding of copy number heterogeneity and the emergence of drug resistance genes in cancer.

## Abstract (precis)

Black et al. demonstrate that hypoxia induces transient, site-specific copy gains in primary, nontransformed, and transformed human cells. Hypoxia-driven copy gains are dependent on the KDM4A histone demethylase and are blocked by inhibition of KDM4A with a small molecule or the natural metabolite succinate.

Cancer is often characterized by copy gains or losses of chromosome arms, whole chromosomes, and/or amplifications/deletions of smaller genomic fragments (Hook et al. 2007; Stratton et al. 2009; Beroukhim et al. 2010). While it has long been understood that tumors within the same pathological subtype have different mutational and copy number profiles (Burrell et al. 2013), it has recently become apparent that intratumoral heterogeneity likely plays an important role in tumor development, metastatic potential, and acquired drug resistance (Gerlinger et al. 2012; Burrell et al. 2013; Junttila and de Sauvage 2013; Nathanson et al. 2014). Traditionally, somatic copy number alterations (SCNAs) and copy number variations (CNVs) have been thought of as heritable genetic events in cancer cells that emerge through an adaptive advantage; however, recent work suggests that at least some copy gains may be transient and could arise given the correct genetic, therapeutic, or environmental conditions (Black et al. 2013; Nathanson et al. 2014). For example, analysis of epidermal growth factor receptor (*EGFR*) mutations and amplifications in glioblastoma patients revealed a transient extrachromosomal amplification of a specific *EGFR* isoform (Nathanson et al. 2014). In addition, amplification and overexpression of the H3K9/36 tridemethylase KDM4A/JMJD2A caused rereplication and transient site-specific copy gains (TSSGs). Furthermore, impairing H3K9 or H3K36 methylation with lysine-to-methionine substitutions (K9M or K36M) resulted in site-specific gains (Black et al. 2013; Lewis et al. 2013). Taken together, these findings suggest that copy gains can be modulated by chromatin changes and selective pressures.

These initial observations highlighted a pathological state that could promote copy gains. However, a major question remained: “Are there physiological signals or cues that cells encounter that in turn cause copy gains within defined regions of the genome?” We reasoned that tumor cells encounter various stresses that could promote copy gains, which could ultimately contribute to the copy number heterogeneity observed in tumors. In fact, we suspect that regions of SCNAs often observed in tumors maybe prone to transient amplification (i.e., 1q12–1q21) and contribute to their observed copy gains in tumors. This same notion could also explain why CNVs of specific regions (e.g., 1q21) emerge in other diseases such as autism and schizophrenia (Stefansson et al. 2008; Levinson et al. 2011).

Therefore, we systematically screened site-specific copy gains after cells were treated with a panel of cellular stresses that occur during development and tumorigenesis. Surprisingly, only one condition, hypoxia, promotes site-specific copy gain of regions frequently observed in tumors. Hypoxia-dependent copy gain occurs at tumor-relevant oxygen levels (1% O_2_) in diverse cancer cell lines and primary T cells. Hypoxia-dependent site-specific copy gains are transient, require S phase, and undergo rereplication. We demonstrate that copy gains were not dependent on HIF1α or HIF2α; however, the α-ketoglutarate-dependent lysine demethylase KDM4A was required for the copy gains. Upon hypoxic exposure, KDM4A was stabilized through reduced association with the SKP1–Cul1–F-box (SCF) ubiquitin ligase complex, increased association with chromatin, and retained enzyme activity. Finally, pretreatment of cells with succinate (a naturally occurring metabolite that inactivates α-ketoglutarate-dependent enzymes) or a lysine demethylase (KDM) chemical inhibitor blocks hypoxia-induced gains. These observations highlight the dynamics associated with copy gain and suggest that enzyme levels, S-phase status, cellular stresses, and metabolic state could contribute to the copy number heterogeneity observed in human tumors.

We demonstrated that, consistent with hypoxia-induced copy gains being a biological response, copy gain following hypoxia is conserved at a syntenic region in zebrafish cells, while a nonsyntenic region was not gained. In addition, primary breast and lung tumors with a defined hypoxic gene signature are enriched for focal copy number changes in the same regions generated in human and zebrafish cell cultures. Most importantly, our analyses of hypoxic breast and lung tumors identified increased copy number and expression of a drug resistance oncogene, *CKS1B* (Shaughnessy 2005). We further demonstrated in breast cancer cells that *CKS1B* exhibited site-specific copy gain and had increased expression upon hypoxic exposure. These results suggest that hypoxia can promote site-specific copy gain and increased expression of drug resistance genes such as *CKS1B*. These data uncover a mechanism that could account for both copy number and expression heterogeneity observed in solid tumors and establish a molecular basis for drug resistance gene selection (Patel et al. 2014).

## Results

### Hypoxia promotes site-specific copy gain

**Figure 1.**
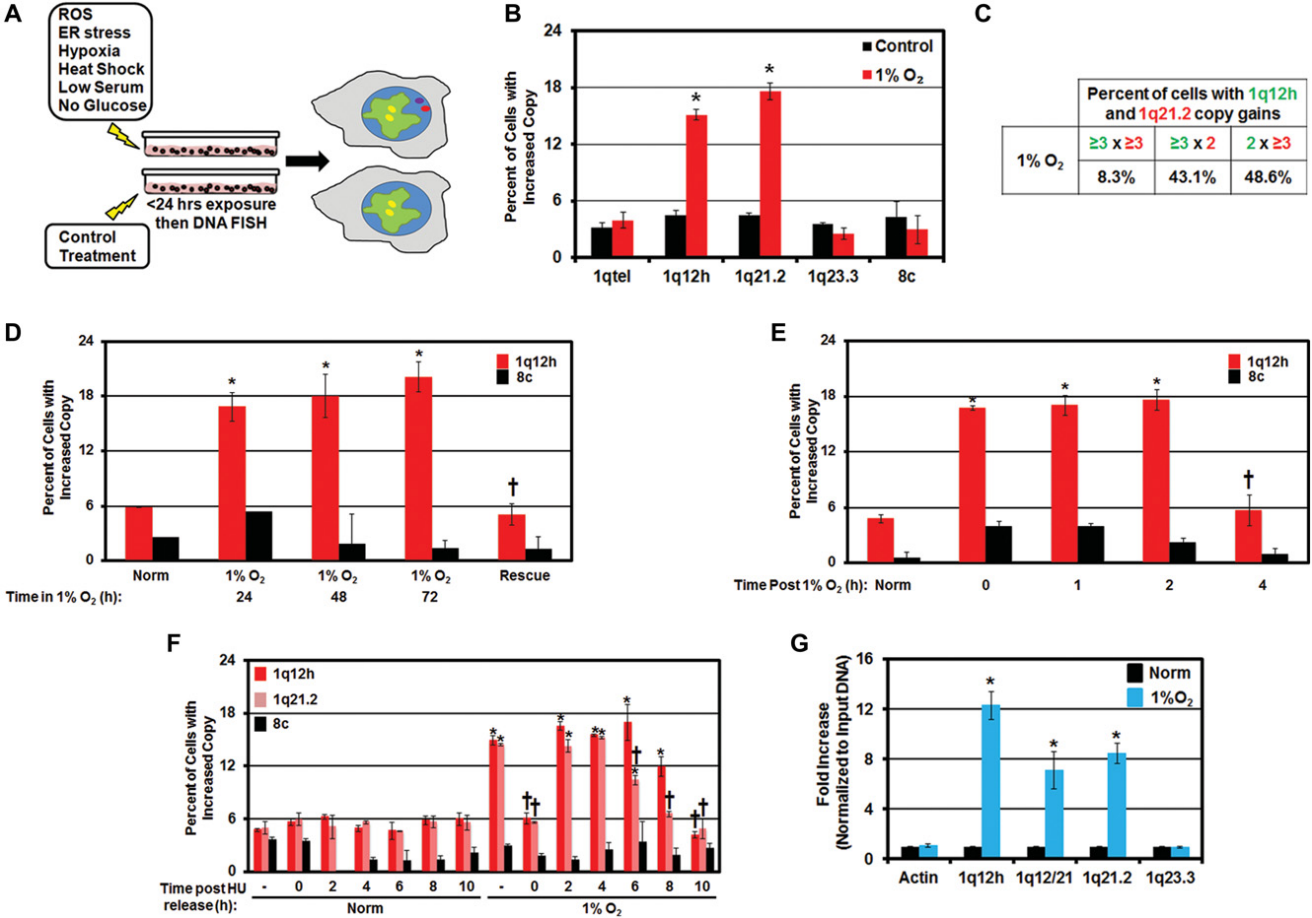
Hypoxia, but not other physiological stresses, promotes TSSG. (*A*) Schematic detailing the approach used in the screen of physiological stresses. RPE cells were exposed to the indicated stress for 24 h prior to collection for FISH and fluorescence-activated cell sorting (FACS) analysis. (*B*) Hypoxia promotes site-specific copy gain of 1q12h and 1q21.2 by FISH analysis. (*C*) Hypoxia-amplified regions are not contiguous. Table summarizing coamplification of 1q12h and 1q21.2. Data are presented as a percentage of all amplified cells (sum of all replicates) having two or three or more (three plus) copies of the indicated FISH probes. (*D*) Hypoxia-induced copy gain of 1q12h is reversible. Quantification of FISH for 1q12h and chromosome 8 centromere (8c) after 24–72 h of 21% O_2_ (normoxia) or 1% O_2_ (hypoxia) or a return to normoxia from 1% O_2_ for 24 h (Rescue). (†) Significant difference from 1% O_2_ for 72 h by two-tailed Student's *t*-test. *P* < 0.05. (*E*) Hypoxia-dependent copy gains are removed within 4 h of return to normoxia. Quantification of FISH probes for the indicated times after 48 h of normoxia or hypoxia treatment. (†) Significant difference from 0 h after release from 1% O_2_ by two-tailed Student's *t*-test. *P* < 0.05. (*F*) Hypoxia-induced copy gains occur during S phase. Quantification of FISH for 1q12h, 1q21.2, and 8c in RPE cells following hydroxyurea (HU) arrest in normoxia or 1% O_2_ (time 0) or the indicated time after HU release. (†) Significant difference from asynchronous (−) 1% O_2_ by two-tailed Student's *t*-test. *P* < 0.05. (*G*) Regions with hypoxia-dependent copy gain are rereplicated. Cesium chloride (CsCl) density gradient purification of rereplicated DNA was analyzed by quantitative PCR (qPCR) for regions amplified in hypoxia. Error bars represent the SEM. (*) Significant difference from normoxia by two-tailed Student's *t*-test. *P* < 0.05.

We reasoned that tumor cells experience various stresses that promote copy gains, which could ultimately contribute to the copy number heterogeneity observed in tumors. Therefore, we monitored the impact that environmental conditions observed during development and tumorigenesis have on regions frequently gained in tumors and susceptible to TSSGs (i.e., 1q12–1q21) (Beroukhim et al. 2010; Black et al. 2013; Tang and Amon 2013). Specifically, we screened copy gain in the nearly diploid, immortalized but nontransformed hTERT-RPE-1 (hereafter called RPE) cell line (Jiang et al. 1999; Black et al. 2013) that was exposed to reactive oxygen species (ROS; H_2_O_2_), ER stress (tunicamycin [TU]), temperature stress (heat shock, 43°C), metabolic stress (low serum [0.1% FBS], no glucose), and hypoxia (1% O_2_) (Fig. 1A; Supplemental Fig. S1A–M). We exposed cells to the indicated stresses (see the Materials and Methods) and assayed for site-specific copy gain by fluorescent in situ hybridization (FISH) and cell cycle profiles after 24 h (Fig. 1B; Supplemental Fig. S1B–M). Using our approach, only hypoxia generated site-specific gains (Fig. 1B), while other stresses were not drastically different from control conditions (Supplemental Fig. S1C–M). For example, 1q12h and 1q21.2 copy gains were induced in as little as 24 h of hypoxic exposure; however, no change was observed for other chromosomal regions (e.g., 1q23.3) (Fig. 1B). Since hypoxic exposure alters the redox state of the cell (Solaini et al. 2010), we examined whether other redox modulators impacted copy gain. Cells exposed to other reducing (DTT and N-acetyl cysteine [NAC]) or oxidizing (2,3-dimethoxy-1,4-naphthoquinone [DMNQ]) agents did not induce site-specific copy gain, suggesting that the observed gains are specific to hypoxia (Supplemental Fig. S1N–S). Spectral karyotyping analysis of hypoxic cells did not show widespread genome instability (Supplemental Table S1), which was consistent with the normal cell cycle profiles observed in hypoxia (Supplemental Fig. S1H). Furthermore, analysis of 1q12h and 1q21.2 in the same cells revealed that the gains in hypoxic conditions were predominantly mutually exclusive (Fig. 1C), which further underscored the site-specific nature of the gains. These results suggest that hypoxia promotes site-specific copy gain.

To address whether copy gain was a prevalent response to hypoxia, we analyzed a diverse panel of cancer cell lines—including breast cancer (MDA-MB 468 and MDA-MB 231), neuroblastoma (SK-N-AS), and multiple myeloma (MM.1S)—and kidney cell lines (HEK293T [hereafter called 293T] and UMRC2) for copy gain of 1q12h by FISH following growth in hypoxia (Supplemental Fig. S2A–M). In each cell line, we observed copy gain of 1q12h under hypoxic conditions but no change in chromosome 8 centromere (8c). Furthermore, HIF1α or HIF2α depletion by two independent siRNAs did not prevent copy gain in hypoxic RPE cells despite blocking induction of the hypoxia-inducible target gene CAIX (Supplemental Fig. S2N–Q). Consistent with these observations, UMRC2 cells—which lack *VHL* and have a functionally stable HIF1α and HIF2α (Gameiro et al. 2013), resulting in hypoxia gene program activation in normoxic conditions—do not generate copy gain without hypoxia (Supplemental Fig. S2K–M). Therefore, HIF1α and HIF2α stabilization was not sufficient to promote copy gain. Together, these data strongly suggest that hypoxia-dependent copy gains are a common response that does not require the HIF1/2α pathway.

### Hypoxia-induced copy gains require proliferation

Oxygen levels change during development and tumorigenesis (Vaupel 2004; Dunwoodie 2009); therefore, we assessed whether site-specific copy gains are reversible upon return to normal oxygen levels (Fig. 1D). FISH analysis for 1q12h copy gain revealed an increased percentage of cells with copy gain at 24, 48, and 72 h of growth in hypoxia; however, upon return to normoxia, the number of cells with extra copies of 1q12h returned to baseline (Fig. 1D). In fact, copy gain of 1q12h persists for the first 2 h following release from hypoxia but is lost by 4 h after return to normoxia (Fig. 1E). These data suggest that hypoxia-dependent copy gains are dynamic and reversible.

To demonstrate that hypoxia-dependent copy gains require proliferation, we arrested cells using hydroxyurea (HU) under hypoxic conditions (Supplemental Fig. S2R). Cells arrested at G1/S in hypoxia did not exhibit copy gains (Fig. 1F). However, upon release from the arrest, hypoxic cells rapidly accumulated copy gain of both 1q12h and 1q21.2. Intriguingly, these gains were lost prior to the end of S phase, with loss of 1q21.2 copy gains occurring slightly faster than 1q12h loss, suggesting that individual regions exhibit site-specific copy gain with different kinetics. Furthermore, we demonstrated that these regions were rereplicated by performing quantitative PCR (qPCR) on DNA purified from the heavy:heavy (H:H) fraction from a cesium chloride (CsCl) density gradient (Fig. 1G; Supplemental Fig. S2S). These results demonstrate that hypoxia-induced copy gains occur during S phase and are reversible.

**Figure 2.**
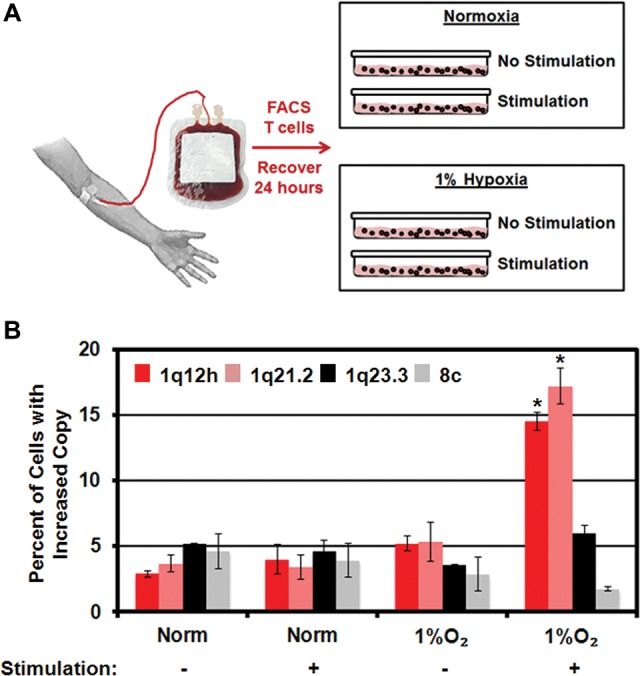
Hypoxia induces site-specific copy in primary human T cells. (*A*) Schematic illustrating collection, isolation, and stimulation of primary human T cells. (*B*) Hypoxia induces site-specific copy gain only in stimulated primary human T cells. Error bars represent the SEM. (*) Significant difference from normoxia by two-tailed Student's *t*-test. *P* < 0.05.

The next major question was whether a hypoxic signal could drive site-specific copy alterations in nonimmortalized or noncancer cells. To address this question, we isolated CD4^+^ T cells by fluorescence-activated cell sorting (FACS) from buffy coat and peripheral blood of healthy individuals (Fig. 2A). Following isolation, T cells were allowed to recover in normoxia (i.e., 21% O_2_, which is “normoxia” for cell culture, similar to the 13.2% O_2_ observed in arterial blood not associated with hemoglobin) (Carreau et al. 2011) for 24 h in the presence of IL2 with or without stimulation with anti-human CD3 and CD28 antibodies. Following recovery, T cells were maintained in normoxia or transferred to hypoxia for an additional 24 h and analyzed by FISH for site-specific copy gain. Only stimulated T cells grown in hypoxia for 24 h exhibited copy gain of 1q12h and 1q21.2 but not gain of 1q23.3 or 8c (Fig. 2B). These results demonstrate that primary cells subjected to hypoxic conditions promote site-specific copy gain in a proliferation-dependent manner.

### KDM4A stabilization promotes hypoxia-induced copy gain

**Figure 3.**
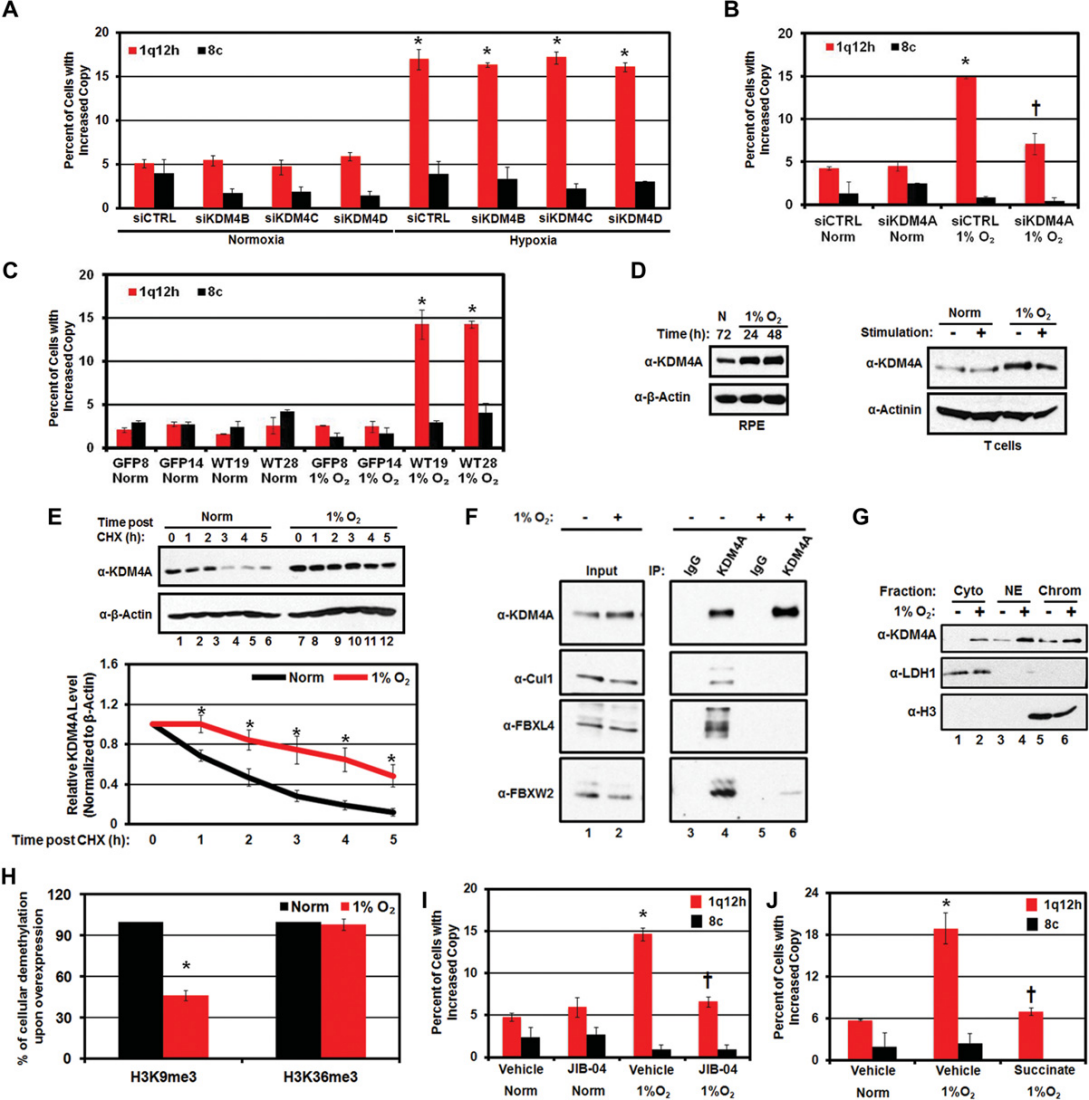
Hypoxia-induced site-specific copy gains are KDM4A-dependent. (*A*) KDM4B–D are not required for copy gain in hypoxia. Quantification of FISH for 1q12h and 8c in RPE cells depleted of KDM4B, KDM4C, or KDM4D and maintained in normoxia or hypoxia. Data presented are an average of two independent experiments, each performed with two independent siRNAs. (*B*) Hypoxia-induced 1q12h and 1q21.2 copy gains require KDM4A. Quantification of FISH for 1q12h and 8c in RPE cells after 24 h of normoxia or hypoxia and with or without depletion of KDM4A. Data presented are an average of two independent siRNA. (*C*) Genomic deletion of *KDM4A* using CRISPR/Cas9 abrogates hypoxia-driven copy gain. Quantification of FISH for 1q12h and 8c in 293T CRISPR cell lines stably expressing either GFP or GFP-KDM4A following 24 h of normoxia or hypoxia. Data represent an average of two independent experiments for two independently derived single-cell clones of GFP (GFP8 and GFP14) or GFP-KDM4A (WT19 and WT28). (*D*) Hypoxia stabilizes KDM4A protein levels. Western blot indicates KDM4A protein levels after 24 and 48 h of hypoxic treatment in RPE cells (*left* panel) and primary human T cells with or without stimulation (*right* panel). (*E*) Hypoxia increases the half-life of KDM4A protein in RPE cells. (*Top* panel) Western blot of half-life experiment demonstrates that KDM4A is stabilized in hypoxia following cycloheximide treatment. (*Bottom* panel) Graphical representation of KDM4A half-life in RPE cells. Quantification of half-life indicates a half-life of 1 h 49 min ± 3 min in normoxia and 4 h 56 min ± 37 min in hypoxia. (*) Significant difference from normoxia at the same time point by two-tailed Student's *t*-test. *P* < 0.05. (*F*) Hypoxia abrogates the interaction of the SCF complex with KDM4A. KDM4A was immunoprecipitated from RPE cells maintained in normoxia or hypoxia, and the interaction with components of the SCF complex was analyzed by Western blot. (*G*) KDM4A levels are increased on chromatin during hypoxia (lanes *5*,*6* , respectively; 1% O_2_). (Cyto) Cytoplasm; (NE) nuclear extract; (Chrom) chromatin fraction. (*H*) KDM4A demethylase activity is retained after 24 h in hypoxia. RPE cells expressing 3xHA-WT-KDM4A were maintained in normoxia or hypoxia for 24 h, and H3K9 and H3K36 demethylation was assessed by immunofluorescence. The graph represents an average of two independent experiments, with demethylase activity in hypoxia normalized to activity in normoxia. (*I*) Demethylase inhibition with JIB-04 blocks hypoxia-dependent copy gain. Quantification of FISH for 1q12h and 8c in RPE cells upon JIB-04 treatment. (*J*) Hypoxia-dependent copy gains can be suppressed by treatment with 2 mM succinate. In all panels, error bars indicate SEM. (*) Significant difference from normoxia (*B*,*C*) and significant difference from vehicle-treated normoxia samples (*I*,*J*) by two-tailed Student's *t*-test. *P* < 0.05. (†) Significant difference from siCTRL (1% O_2_; *B*) and significant difference from vehicle (1% O_2_; *I*,*J*) by two-tailed Student's *t*-test. *P* < 0.05.

Since our previous study demonstrated that depletion of either H3K9me3 or H3K36me3 was sufficient to promote site-specific copy gain (Black et al. 2013), we reasoned that histone demethylases may mediate hypoxia-induced copy gain. JmjC-containing demethylases use molecular oxygen as a cofactor for demethylation, and thus hypoxia has been proposed to inactivate the JmjC-containing demethylases. However, previous reports have shown that certain JmjC-containing KDMs that target H3K9 methylation are transcriptionally up-regulated (KDM4B and KDM4C) or retain their activity (KDM3A) upon hypoxic exposure (Krieg et al. 2010; Lee et al. 2013). We tested whether KDM3A overexpression or siRNA depletion of KDM4 enzymes with independent siRNAs during hypoxia was responsible for site-specific gain. KDM3A overexpression was not sufficient to promote site-specific copy gain (Supplemental Fig. S3A,B). In addition, depletion of KDM4B–D with two independent siRNAs did not block hypoxia-induced copy gain despite increased KDM4B/C expression in hypoxia (Fig. 3A; Supplemental Fig. S3C–F). However, depletion of KDM4A blocked the hypoxia-dependent copy gain (Fig. 3B; Supplemental Fig. S3G) without altering cell cycle distribution (Supplemental Fig. S3H).

To demonstrate a genetic requirement for KDM4A, we generated *KDM4A* knockout 293T cells using CRISPR/Cas9. We then reintroduced either *GFP* or *GFP-KDM4A* (wild type) and generated single-cell clones. We selected GFP-KDM4A clones that had expression levels similar to those of endogenous KDM4A in parental 293T cells (Supplemental Fig. S3I). Importantly, the restored GFP-KDM4A was induced under hypoxic conditions (Supplemental Fig. S3J). Two independent GFP clones (lacking endogenous KDM4A) were unable to generate site-specific copy gain in hypoxia, while both GFP-KDM4A rescue clones were able to generate site-specific copy gains (Fig. 3C) without altering cell cycle distribution (Supplemental Fig. S3K). These results demonstrate that KDM4A is necessary for the generation of site-specific copy gain in response to hypoxia.

In agreement with previous reports, we did not observe increased KDM4A transcript upon hypoxic exposure (Supplemental Fig. S3L; Beyer et al. 2008). However, KDM4A protein levels were increased with as little as 24 h of exposure to hypoxia in all cell lines tested (Fig. 3D, left panel; Supplemental Fig. S3M) as well as in the primary CD4^+^ T cells treated with hypoxia (Fig. 3D, right panel). In fact, KDM4A protein levels were regulated in the same temporal fashion as the copy gains upon hypoxic exposure and return to normoxia (Supplemental Fig. S4A–C). Furthermore, hypoxia resulted in KDM4A protein stabilization (e.g., increased half-life from 1 h 49 min to 4 h 56 min) (Fig. 3E; Supplemental Fig. S4D). We and others previously demonstrated that KDM4A proteins levels are regulated by the SCF-containing ubiquitin ligase complex (Tan et al. 2011; Van Rechem et al. 2011). KDM4A interacts with the SCF–ubiquitin ligase complex and is ubiquitinated and degraded in a cell cycle-dependent manner. Therefore, we reasoned that this complex may influence KDM4A ubiquitination and protein stability during hypoxia exposure. Consistent with our previous results and the increased half-life of KDM4A in hypoxia, KDM4A had a reduced association with the SCF complex and less ubiquitination under hypoxic conditions (Fig. 3F; Supplemental Fig. S4E,F; Van Rechem et al. 2011).

We previously demonstrated that KDM4A overexpression results in increased chromatin association throughout the genome and is associated with rereplication of specific regions (Van Rechem et al. 2011; Black et al. 2013). In agreement with these observations, hypoxia resulted in stabilized KDM4A that also increased in the chromatin fraction (Fig. 3G). To determine whether KDM4A remained active under hypoxic conditions, we assessed demethylation using standard immunofluorescence assays (Whetstine et al. 2006). Importantly, KDM4A retained enzymatic activity under hypoxic conditions. Twenty-four-hour exposure to hypoxic conditions resulted in a reduction but not a loss in H3K9me3 activity while not affecting H3K36me3 demethylation (Fig. 3H; Supplemental Fig. S4G). KDM4A remained active, with a modest reduction in demethylase activity, even after 48 h in hypoxic conditions (Supplemental Fig. S4H). These results demonstrate that KDM4A was stabilized, enriched on the chromatin, and able to retain enzymatic activity under hypoxic conditions.

### Small molecule inhibition of hypoxia-induced copy gains

Based on these observations, we hypothesized that KDM4A inhibition could serve as a tool to modulate the copy number alterations observed in hypoxia. To test this hypothesis, we pretreated cells with an inhibitor of JmjC demethylases, JIB-04 (Wang et al. 2013; Van Rechem et al. 2015). JIB-04 is not a selective inhibitor of KDM4A but inhibits the KDM4 family as well as KDM5A and KDM6B (Wang et al. 2013). JIB-04 did not substantially alter KDM4A protein levels or cell cycle profiles in hypoxia (Supplemental Fig. S4I,J). However, treatment with JIB-04 significantly reduced hypoxia-dependent copy gain of 1q12h (Fig. 3I). Since JIB-04 also targets KDM5A and KDM6B, we depleted these KDMs with siRNAs under hypoxic conditions. Depletion of KDM5A or KDM6B was insufficient to rescue hypoxic induction of site-specific copy gain (Supplemental Fig. S4K–N). Since depletion of KDM4B–D, KDM5A, or KDM6B failed to rescue site-specific copy gain in hypoxia, JIB-04 is likely suppressing site-specific gain through KDM4A inhibition.

Since all JmjC-containing proteins can be inhibited by the natural metabolite succinate (Smith et al. 2007; Black et al. 2012), we treated RPE cells with succinate prior to growth in hypoxia. Succinate treatment did not alter KDM4A stabilization or cell cycle progression (Supplemental Fig. S4O,P) but was sufficient to abrogate hypoxia-dependent copy gain of 1q12h (Fig. 3J). These results establish that hypoxia-dependent copy gains are a biological response that can be pharmacologically regulated and emphasize the impact that metabolic state can have on copy number. In addition, these data illustrate how a metabolic change could counteract hypoxia-induced gains, which provides another basis for copy number heterogeneity within tumors (see Fig. 7, below).

### Hypoxia-induced copy gains are conserved

**Figure 4.**
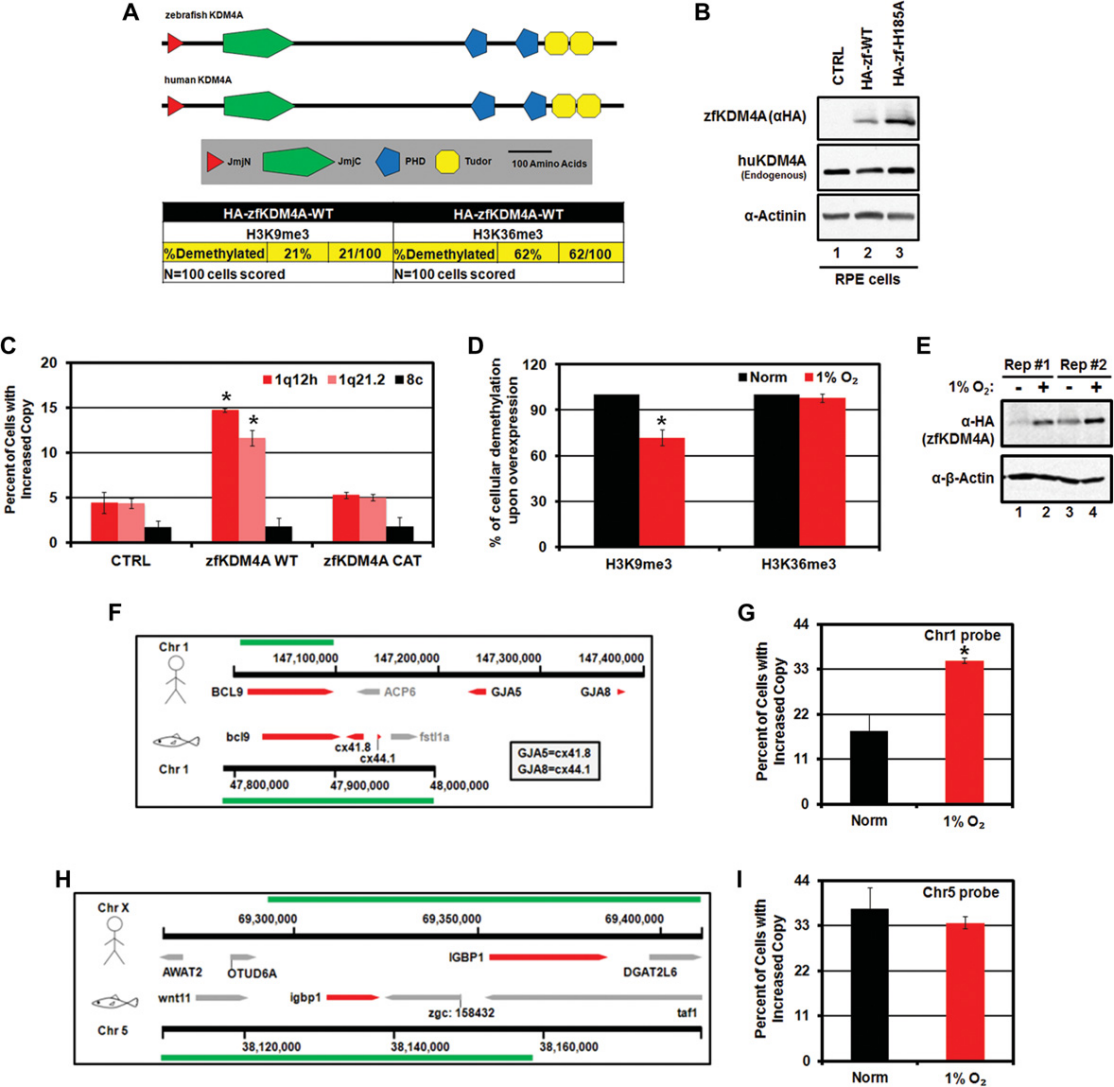
Hypoxia-induced copy gains are conserved in zebrafish. (*A*) A schematic depicting the homology of huKDM4A and zfKDM4A. The table depicts the H3K9 and H3K36 demethylase activity of zfKDM4A expressed in RPE cells as determined by immunofluorescence. (*B*) Expression levels of zfKDM4A and huKDM4A proteins in RPE cells expressing wild-type (WT) and catalytically mutant (H185A) zfKDM4A. (*C*) zfKDM4A promotes copy gain in human cells. Quantification of FISH for 1q12h, 1q21.2, and 8c for RPE cells expressing zfKDM4A or catalytically inactive zfKDM4A CAT. (*D*) Quantification of H3K9 and H3K36 demethylase activity by immunofluorescence in normoxia and hypoxia for RPE cells ectopically expressing zfKDM4A. (*E*) Hypoxia stabilizes zfKDM4A in RPE cells. (*F*) A schematic depicting the syntenic region of 1q21.2 in zebrafish used for FISH analysis. Green bars indicate the location of the human (stick figure) and zebrafish (fish icon) probes used. (*G*) Hypoxia promotes copy gain of *BCL9* in zebrafish AB.9 cells. Quantification of FISH for *BCL9* after 72 h of normoxia or 1% O_2_. (*H*) A schematic of the *IGBP1* homologous region in zebrafish. Green bars indicate the location of the human (stick figure) and zebrafish (fish icon) probes used. (*I*) Hypoxia does not induce copy gain of *IGBP1* in zebrafish. Quantification of FISH for *IGBP1* after 72 h of normoxia or 1% O_2_. Error bars represent the SEM. (*) Significant difference from control samples by two-tailed Student's *t*-test. *P* < 0.05.

Based on our findings in primary and cancer cells, we hypothesized that hypoxia-induced KDM4A stabilization and copy gains were evolutionarily conserved responses. In order to test this possibility, we examined zebrafish KDM4A (zfKDM4A). Wild-type zfKDM4A (zfKDM4A-WT), which has an architecture similar to that of human KDM4A (huKDM4A) (Fig. 4A), was able to demethylate both H3K9me3 and H3K36me3 (Fig. 4A). In addition, overexpression of the catalytically active zfKDM4A (zfKMD4A-WT) in human cells was sufficient to promote copy gain of regions regulated by huKDM4A (Fig. 4B,C). Similar to huKDM4A, zfKDM4A retained catalytic activity in hypoxia, albeit with reduced activity on H3K9me3, and was stabilized under hypoxic treatment (Fig. 4D,E).

This prompted us to evaluate whether hypoxia promoted copy gain in zebrafish cells. Zebrafish were cultured in water saturated with atmospheric oxygen levels (21%), and zebrafish cell lines were considered to be hypoxic at or below 3% O_2_ (Jopling et al. 2012). Using the zebrafish cell line AB.9 (Paw and Zon 1999), we assessed the ability of hypoxia to promote copy gain of a region syntenic to the human *BCL9* gene on 1q21.2 (Fig. 4F). This syntenic region was gained in AB.9 cells upon hypoxia exposure (Fig. 4G). However, a second homologous but nonsyntenic region to the human *IGBP1* gene on zebrafish chromosome 5 (Fig. 4H, region covered by the Xq13.1 probe in human cells, green bar at the top of the schematic) was not copy-gained in response to growth in hypoxia (Fig. 4I). These data demonstrate that copy gain is a conserved response to hypoxia.

### Hypoxic tumors are enriched for hypoxia-induced copy gains

Since primary cells, cultured cancer lines, and zebrafish cells promote site-specific gain in response to hypoxia, we hypothesized that hypoxic conditions within primary tumors may contribute to SCNAs observed in tumors (Beroukhim et al. 2010). By analyzing tumors, we are controlling for the physiological hypoxia that is occurring within the tumor. This analysis circumvents the issue of our standard cell culture conditions (21% O_2_, normoxia in vitro) and establish whether the relationship that we observed in culture is occurring in tumors. Ultimately, this analysis will allow in vivo validation and in turn allow us to test newly identified regions in cell culture models.

**Figure 5.**
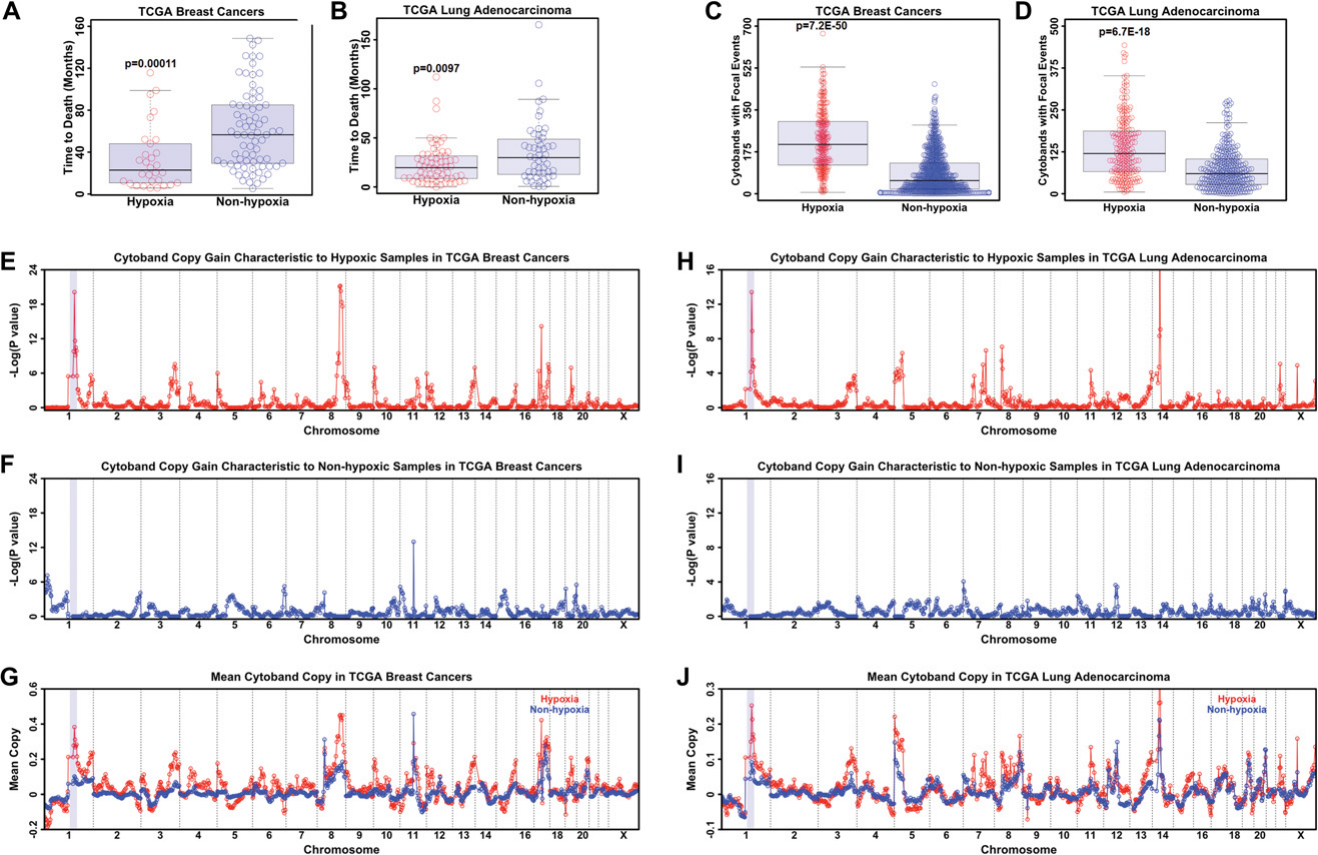
Tumors with a hypoxic signature have copy gains of regions observed in hypoxic cell culture. (*A*) TCGA breast cancer samples with a hypoxic gene signature have a faster time to death. (*B*) TCGA lung adenocarcinoma samples with a hypoxic gene signature have a faster time to death. (*C*) TCGA breast cancer samples with a hypoxic gene signature have increased focal CNVs. (*D*) TCGA lung adenocarcinoma samples with a hypoxic gene signature have increased focal CNVs. (*E*) TCGA breast cancer samples with a hypoxic gene signature have an enrichment of copy gain of 1p11.2 through 1q23.3. (*F*) TCGA breast cancer samples without a hypoxic gene signature do not have enrichment of copy gain of 1p11.2 through 1q23.3. (*G*) Mean copy number of hypoxic (red) and nonhypoxic (blue) breast cancer samples. (*H*) TCGA lung adenocarcinoma samples with a hypoxic gene signature have enriched copy gain of 1p11.2 through 1q23.3. (*I*) TCGA lung adenocarcinoma samples without a hypoxic gene signature do not have enriched copy gain of 1p11.2 through 1q23.3. (*J*) Mean copy number of hypoxic (red) and nonhypoxic (blue) lung adenocarcinoma samples. For each coamplification plot, blue shaded regions indicate 1p11.2 through 1q23.3.

To address our hypothesis, we analyzed primary breast (BRCA) and lung (LUAD) tumors from The Cancer Genome Atlas (TCGA) for SCNAs in hypoxic compared with nonhypoxic tumors. To identify hypoxic tumors, we used the hypoxia gene signature derived by Winter et al. (2007) to perform an unbiased consensus hierarchical clustering of BRCA and LUAD (see “Data Processing for TCGA Breast Cancer and Lung Adenocarcinoma” in the Materials and Methods; Supplemental Fig. S5A–D; The Cancer Genome Atlas Network 2012). As validation of this gene set and clustering approach, 65 out of 88 basal BRCA samples reside in the hypoxic cluster. Basal breast cancer had been previously demonstrated to be more hypoxic than other molecular subtypes of breast cancer, which supports our computational analyses (Perou 2010). Furthermore, previous reports had demonstrated that hypoxia is a negative prognostic marker in multiple tumor types (Hockel et al. 1996; Eschmann et al. 2005; Wang et al. 2014). Our analyses further substantiated these observations, since hypoxic BRCA and LUAD samples had a significantly higher risk (faster time to death) in both BRCA (Fig. 5A) and LUAD (Fig. 5B).

We next asked whether specific cytogenetic bands exhibit focal amplifications in hypoxic BRCA and LUAD samples. In fact, BRCA and LUAD samples also had an increased number of focal copy number events in hypoxic samples (Fig. 5C,D; Supplemental Fig. S5E–H). We observed a strong enrichment of copy gain of 1p11.2 through 1q23.3 (Fig. 5E,H, blue shaded region) in hypoxic BRCA (Fig. 5E) and LUAD (Fig. 5H) that was not present in nonhypoxic samples (Fig. 5F–J). Taken together, our data highlight that hypoxic conditions are associated with a worse outcome and focal SCNAs in tumors and that regions with hypoxia-dependent copy gain in cell culture are also focally gained in hypoxic primary tumors in two different cancer types. These data further emphasize the relationship between hypoxia and driving site-specific copy gain in vitro and in vivo.

### Hypoxia induces copy gain and expression of a drug-resistant oncogene

**Figure 6.**
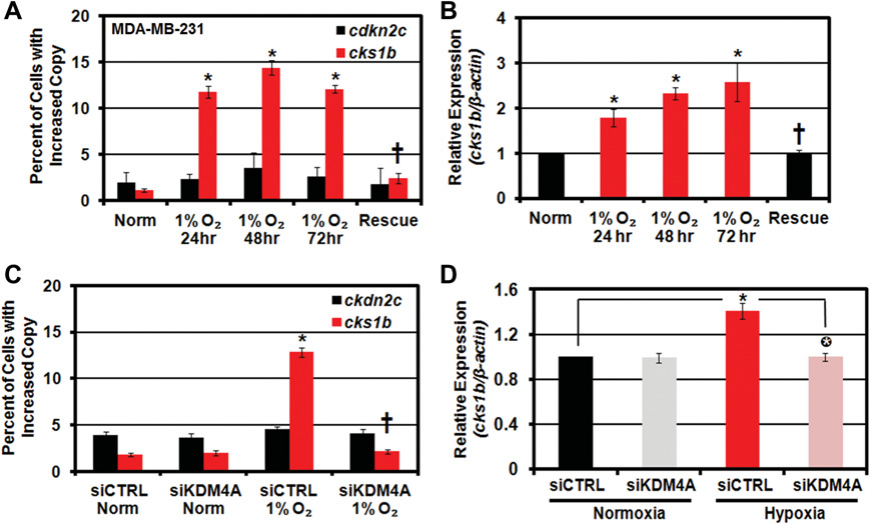
*CKS1B* exhibits site-specific copy gain and increased expression in hypoxic cells. (*A*,*B*) *CKS1B* is copy-gained and overexpressed in hypoxic breast cancer cell lines. Quantification of FISH (*A*) and CKS1B mRNA expression (*B*) in MDA-MB 231 cells maintained in hypoxia for 24–72 h or maintained in hypoxia for 48 h prior to return to normoxia for 24 h (rescue). (†) Significant difference from 1% O_2_ at 24 h by two-tailed Student's *t*-test. *P* < 0.05. (*C*) Hypoxia-dependent *CKS1B* copy gain requires KDM4A. Quantification of FISH for 1q12h and 8c for MDA-MB-231 cells maintained in normoxia or hypoxia with or without siRNA depletion of KDM4A. (†) Significant difference from 1% O_2_ siCTRL by two-tailed Student's *t*-test. (*D*) Hypoxia-dependent CKS1B transcript induction requires KDM4A. Circled asterisks indicate significant difference from siCTRL in hypoxia by two-tailed Student's *t*-test. *P* < 0.05. In all panels, asterisks indicate significant difference from normoxia by two-tailed Student's *t*-test. *P* < 0.05.

To date, a function for TSSGs has yet to be assigned. Therefore, we asked whether hypoxic exposure served as a mechanism to promote gene amplification and in turn increase gene expression. Analysis of both BRCA and LUAD identified seven genes that were amplified and had altered expression in both tumor types (Supplemental Table S2). Of particular interest was the drug resistance oncogene *CKS1B*, which has low-level copy gains (one to three copies) in several cancers (Shaughnessy 2005). This level of gain corresponds to a comparable increased expression in tumors, which is associated with drug resistance and worse outcome in patients (Wang et al. 2009; Shi et al. 2010; Martin-Ezquerra et al. 2011; Khattar and Thottassery 2013). Since this target emerged from our in silico analyses and has major implications in tumor drug response and patient outcome, we determined whether *CKS1B* was copy-gained. Using the breast cancer line MDA-MB-231, we observed copy gain for *CKS1B* upon hypoxic exposure, which was reversed upon return to normoxia (Fig. 6A). The gain of *CKS1B* also correlated with an increase in transcription of *CKS1B*, which was rescued upon returning the cells to normoxia (Fig. 6B). We further demonstrated that KDM4A depletion was sufficient to block both the copy gain and transcriptional increase observed for *CKS1B* under hypoxic conditions (Fig. 6C,D; Supplemental Fig. S5I). Taken together, these results suggest that hypoxia can promote site-specific copy gain and increased expression of drug resistance genes such as *CKS1B*. These data uncover a mechanism that could account for both copy number and expression heterogeneity observed in solid tumors (Patel et al. 2014).

## Discussion

We identified a cellular mechanism of TSSGs in response to hypoxic stress. This mechanism does not require genetic manipulation or drug treatment. Cells exposed to tumor-relevant hypoxia (1% O_2_) (Rofstad 2000), but not other physiological stresses, exhibited copy gain in as little as 24 h. Hypoxia promoted site-specific gains in not only transformed cancer cells but also primary human T cells. The generation of site-specific copy gains was conserved across species, as a syntenic region in zebrafish cells was also gained when exposed to hypoxia. Analysis of primary human tumors from TCGA demonstrated that breast and lung tumors that exhibit a hypoxic gene signature were associated with copy gains in the regions generated in human and zebrafish cell cultures. Most importantly, we demonstrated that hypoxic tumors predicted amplification and expression for the drug-resistant oncogene *CKS1B*, which was confirmed in a human breast cancer cell line treated with hypoxia. These copy gains were the result of KDM4A stabilization, which was reversible upon normoxia exposure. We further demonstrated that hypoxia-dependent copy gains are druggable, as pretreatment of cells with succinate or a KDM chemical inhibitor blocked hypoxia-induced copy gains. Taken together, our work uncovered a conserved response to hypoxia from zebrafish to humans that generates site-specific copy gains. These results also highlight how hypoxia could contribute to tumor heterogeneity and suggest that KDM4A inhibitors may be useful cotherapeutics to suppress copy gains.

**Figure 7.**
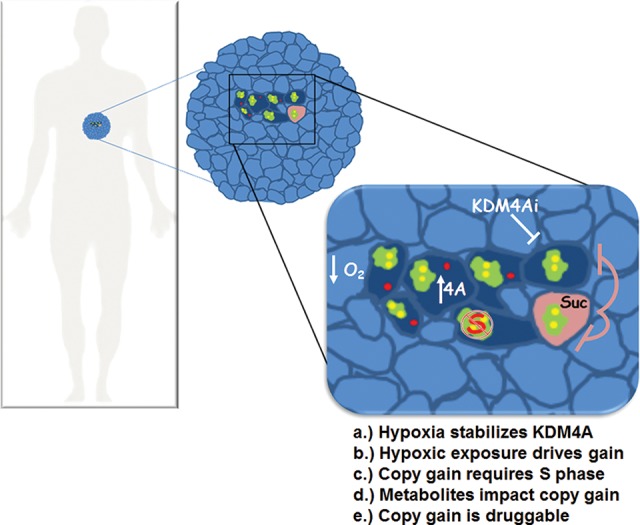
Model depicting how site-specific copy gains could explain intratumoral heterogeneity.

This study provides a mechanistic view of how tumors could acquire intratumoral heterogeneity and how variations in copy number could arise during tumor development. Our work also suggests that intratumoral heterogeneity could include not only stable, heritable SCNA from different subclones but also transient heterogeneity arising from environmental factors, changes in cell cycle, metabolism, or altered chromatin state. Furthermore, our results underscore how nongenetic alterations in the tumor microenvironment, including the availability of oxygen or metabolites (i.e., succinate), could contribute to or limit intratumoral heterogeneity (Fig. 7; Junttila and de Sauvage 2013). These findings highlight the conserved impact that stress, metabolic state, and proliferative capacity could have on intratumor CNV, which has been documented across cancers (Gerlinger et al. 2012; Burrell et al. 2013; Junttila and de Sauvage 2013; Nathanson et al. 2014). It is not yet clear whether this environmental control of copy gain could be tumorigenic under specific circumstances. We view the mechanism described here not as a transforming event but as an adaptive response to stress, which could also serve to modify oncogenic potential. Furthermore, it is possible that the conditions in our screen also promote site-specific copy gain of regions different from those that we tested, require a more prolonged exposure to the stimuli, or arise from an alternative cell of origin. Regardless, we believe that cells control amplification of specific regions of their genome in response to different stimuli to facilitate stress response, survival, and adaptation to new or challenging environmental conditions.

Several KDMs have now been shown to be transcriptionally up-regulated under hypoxic conditions, including KDM4B, KDM4C, and KDM6B (Krieg et al. 2010; Lee et al. 2013; Guo et al. 2015). We observed similar results in RPE cells in response to hypoxia. However, we demonstrated that KDM4A regulation under hypoxic conditions is distinct from these other JmjC KDMs, as it is regulated primarily at the protein level and not at the transcriptional level. We also demonstrate that KDM4A remains active, albeit with reduced activity under hypoxic conditions. The fact that H3K9me3 is more affected after 24 h of hypoxia raises the possibility that hypoxia could also affect substrate specificity. This could be accomplished through post-translational modification of KDM4A or by altering association with a cofactor that may regulate activity. These same post-translational modifications could also be important for altering association of KDM4A with the SCF complex under hypoxic conditions. Identifying what modifications or alterations allow dissociation of KDM4A from the SCF complex will be important and could identify additional pathways that, if misregulated in cancer, could promote TSSGs.

Here, we also demonstrated that hypoxia induces copy gain of a syntenic region to human 1q21.2 in zebrafish cells. Importantly, this reveals that copy gains of related chromosomal domains are conserved across species in response to hypoxia. It is interesting to note that the surrounding gene position and chromosome architecture are conserved between human 1q21.2 and zebrafish *BCL9*, indicating a conserved syntenic structure. In contrast, a zebrafish region homologous to the human Xq13.1 *IGBP1* locus, which was amplified in response to hypoxia in human cells, was not amplified in zebrafish cells. This region did not have a conserved genic or chromosomal architecture and thus was nonsyntenic. This suggests that perhaps syntenic regions or chromosome domains might influence the ability of regions to undergo site-specific copy gains, which is an area that needs further exploration.

The fact that a conserved region amplifies in response to hypoxia in zebrafish and human cells implies that the gained regions may have a function in response to hypoxia. It will be interesting to determine whether these amplified regions are expressed and whether their cognate gene products contribute to hypoxic response during development, which seems possible, since hypoxia induced *CKS1B* copy gain and expression. Alternatively, the fragments themselves may play an important role irrespective of expression, perhaps through priming the DNA damage response. Regardless of the copy gain function, identifying additional regions undergoing copy gain selection and examining their conservation across species will result in important insights into chromosomal organization and syntenic function upon stress or misregulation of KDM4A.

Previous reports have highlighted the selection of regions during cancer progression and development. For example, the *DHFR* gene is amplified upon methotrexate chemotherapy (Alt et al. 1978). In a similar fashion, *EGFR* amplification is lost upon chemotherapy, but extrachromosomal amplification reappears upon drug removal (Nathanson et al. 2014). In addition, several developmentally regulated gene-specific amplifications have been documented, including egg shell gene amplification in *Drosophila* follicle cells, the amplification of genes important for saliva proteins in *Sciara*, and rRNA gene amplification in *Tetrahymena* (Tower 2004). However, the molecular basis for these phenomena has not yet been determined. In the case of cancer, it has been thought to be random selection, while, during development, it has been thought to be a specialized process. The appearance of these specific loci coupled to our most recent findings suggests that specific regulatory factors are involved in the amplification of distinct regions within the genome. Understanding how cells specify these regions and regulate amplification will provide fundamental insights into both developmental and pathological processes. In light of our findings, we predict that the modulation of specific chromatin regulators such as lysine methyltransferases and KDMs will control these events, which will impact development and disease.

In closing, this study documented the conserved role of hypoxia on site-specific copy gains and demonstrated that this process has a molecular basis. While the present study identifies KDM4A as a key enzymatic regulator of this response, it is unlikely to be the only regulator. It will also be interesting to isolate other enzymes that are capable of generating site-specific copy number changes and establish the upstream pathways that could trigger their contribution to copy number alterations. By identifying other conditions and enzymes capable of controlling copy number, we will be able to better understand the genomic diversity that emerges in tumors and during development.

## Materials and methods

### Cell culture and transfections

293T, RPE, MDA-MB 231, MDA-MB 468, and UMRC2 cells were maintained in DMEM with 10% fetal bovine serum (FBS), 1% penicillin/streptomycin, and L-glutamine. SK-N-AS cells were maintained in DMEM/F12 (Gibco) with 10% FBS, 1% penicillin/streptomycin, and L-glutamine. MM.1S cells were maintained in suspension in RPMI with 10% FBS, 1% penicillin/streptomycin, and L-glutamine. Zebrafish AB.9 cells (Paw and Zon 1999) were purchased from American Type Culture Collection and maintained in DMEM with 20% FBS, 1% penicillin/streptomycin, and L-glutamine at 28°C. Transient transfection experiments were performed using Roche X-tremeGENE 9 or Lipofectamine 3000 transfection reagent in OPTI-MEM I medium (Gibco) for 4 h or overnight. No selection was used in transient transfection experiments. siRNA transfections were carried out using Roche X-tremeGENE 9 siRNA reagent or Lipofectamine 3000 in OPTI-MEM I for 4 h or overnight. Each siRNA experiment represents the average of at least two different siRNAs for each target gene.

### Hypoxic conditions

Cells were plated onto culture dishes and allowed to adhere for 20–24 h in normoxia (5% CO_2_, 21% O_2_, 74% N_2_). For hypoxic treatment, cells were maintained in a HERA Cell 150 incubator (Thermo Scientific) flushed with 5% CO_2_ and 1% O_2_ and balanced with N_2_ for the duration of the experiment. Incubator calibrations and verifications were carried out by Bianchi Associates Calibrations/Verifications.

### Drug treatments and synchronization

Cells were treated with the following chemical and metabolic stresses for 24 h at doses used previously: 2 µg/mL TU (Abcam), 60 µM H_2_O_2_ (Thermo Fisher Scientific), reduced-serum DMEM (0.1% FBS), glucose-free DMEM (Gibco), 2 mM DTT (Sigma), 5 mM NAC (Sigma), and 1 µM DMNQ (Sigma). For heat-shock treatment, cells were incubated for 30 min at 43°C and returned for 24 h prior to collection to 37°C .

For G1/S synchronization, cells were treated with 2 mM HU (Sigma) for 20 h. To release, cells were washed twice with culture medium preconditioned in normoxia or hypoxia and supplied with fresh preconditioned medium. For JIB-04 treatment, normoxic cells were pretreated with 62.5 nM JIB-04 (Xcessbio) for 24 h and then treated again with JIB-04 and either transferred to 1% O_2_ or maintained in normoxia for an additional 24 h. Succinate (Sigma, S9637) was administered at a final concentration of 2 mM, and cells were either maintained in normoxia for 72 h or maintained in normoxia for 48 h prior to being transferred to 1% O_2_ for 24 h.

### FISH

FISH was performed as described in Manning et al. (2010) and Black et al. (2013). For RPE cells, copy gain was scored as any cell with three or more distinct foci. Approximately 100 cells for each replicate were scored for all experiments. All FISH experiments include at least two biological replicates. For each experiment, at least one replicate includes FACS and Western blot from the same cells used for FISH. For knockdown experiments, at least two different siRNAs were used for each target. Results are presented as the average from both of the independent siRNAs. Complete methods are in the Supplemental Material.

### Western blots

Western blots were performed as in Black et al. (2010). Complete methods are in the Supplemental Material.

### Expression plasmids and siRNAs

pCS2-3xHA-huKDM4A and pCS2-3xHA-zfKDM4A-WT and catalytic mutants were prepared by gateway transfer into pCS2-3xHA. All clones were sequence-verified. Silencer Select siRNAs were purchased from Life Technologies as follows: KDM4A (s18636, s18637, and s18635), KDM4B (s22867 and s229325), KDM4C (s22989 and s225929), KDM4D (s31266 and s31267), KDM5A (s11834 and S11836), KDM6B (s23109 and s23110), HIF1α (s6539 and s6541), and HIF2α (s4698 and s4700). Results for FISH with each siRNA (at least two independent siRNAs per target) were averaged together in all knockdown experiments presented.

### RNA extraction and qPCR

Cells for RNA isolation were collected by scraping or trypsinization and washed twice with PBS. Cells were resuspended in Tri-Reagent (Roche) and stored at −80°C until use. RNA was isolated using the miRNAeasy Plus kit with on-column DNase digestion (Qiagen) following the manufacturer's instructions and quantified using a Nanodrop 1000D. Single-strand cDNA was prepared using the Transcriptor first strand cDNA synthesis kit (Roche) with oligo dT primers. Expression levels were analyzed by quantitative real-time PCR in a LightCycler 480 with FastStart Universal SYBR Green master (Roche) following the manufacturer's protocols. All samples were normalized by comparison with β-actin transcript, and hypoxia induction was verified with primers for CAIX. For CKS1B transcript analysis, we observed transcript induction in hypoxia in all samples from untreated MDA-MB-231 cells (Fig. 6A). However, transfection of MDA-MB-231 cells reduced the induction level of CKS1B (we considered >1.15-fold induced) (Fig. 6D) and resulted in induction in 16 of 24 replicates, and siKDM4A depletion resulted in reduced CKS1B transcript in 15 of 16 induced replicates. Replicates included three different KDM4A siRNAs. The data represent an average of all replicates that exhibited induction of CKS1B in hypoxia (16 of 24). *CKS1B* was amplified (FISH) in all replicates and not amplified upon KDM4A depletion. Primers are available on request.

### Catalytic activity of huKDM4A and zfKDM4A in hypoxia

Assays for demethylase activity were performed using immunofluorescence as described in Whetstine et al. (2006). Briefly, The indicated HA-tagged KDM4A constructs were transfected into RPE cells grown on coverslips in six-well dishes using X-tremeGENE 9 (Roche) or Lipofectamine 3000 (Life Technologies) DNA transfection reagent. Following 24 or 48 h in hypoxia, H3K36me3 and H3K9me3 were assayed by examining transfected cells (positive for HA staining; Covance, HA.11) following fixation (Whetstine et al. 2006; Black et al. 2013). Approximately 50 highly transfected cells in each of two biological replicates were scored for each condition. Data presented for normoxia are an average of the two replicates. For hypoxia, data are presented as the percentage of activity of the same construct under normoxic conditions for each of two biological replicates, which were averaged together.

### Human CD4^+^ T-cell purification and in vitro culture

CD4+ T cells were isolated from peripheral blood of healthy donors or buffy coats (Sanguine Biosciences) by flow cytometry. Complete isolation and culture conditions are in the Supplemental Material.

### Half-life determination

Protein turnover was assessed as outlined in Van Rechem et al. (2011). Briefly, cells maintained in normoxia and hypoxia were treated with 400 µM cycloheximide (Sigma) for the indicated time, after which lysates were prepared and analyzed by Western blot.

### Immunoprecipitation

Immunoprecipitations were carried out as in Van Rechem et al. (2011) on cells grown in normoxia or hypoxia for 24 h. KDM4A was immunoprecipitated from whole-cell lysates using KDM4A-P006, KDM4A-P014, and KDM4A rabbit polyclonal antibody (Black et al. 2010; Van Rechem et al. 2015). For ubiquitination determination, KDM4A immunoprecipitations were washed under denaturing conditions as in Van Rechem et al. (2011). Ubiquitination of KDM4A was quantitated using ImageJ and normalized to the amount of KDM4A immunoprecipitated.

### CsCl gradient centrifugation

CsCl density gradient centrifugation was performed as in Black et al. (2013). Complete methods are in the Supplemental Material.

### Flow cytometry and cell cycle analysis

Asynchronously growing or G1/S-arrested cells were prepared and fixed as in Black et al. (2010). Cells were stained with 10 µM EdU for 1 h prior to collection. Cell cycle was analyzed by PI staining or EdU incorporation using a Click-IT EdU flow cytometry assay kit (Life Technologies). Flow cytometry of CD4^+^ T cells and cell cycle distribution were analyzed using a BD FACS ARIA II.

### Cell fractionation

Cytoplasmic, nuclear, and chromatin fractions were prepared from RPE cells as described in Van Rechem et al. (2015). Complete methods are in the Supplemental Material.

### Generation of *KDM4A* knockout 293T cells using CRISPR/Cas9

We created *KDM4A* knockout 293T cells as previously described (Fu et al. 2014). Complete methods can be found in the Supplemental Material. We generated genetic rescue lines by reintroducing *GFP* or *GFP-KDM4A*. *KDM4A*-deficient cell lines expressing either GFP or GFP-KDM4A were generated using retroviral infections of pMSCV-GFP or pMSCV-GFP-KDM4A as described in Black et al. (2013). Expression of GFP or GFP-KDM4A was confirmed by Western blot, and no detectable endogenous KDM4A was observed. As clones were derived from 293T cells, clonal variability for chromosome numbers was observed (i.e., chromosome 1). The independent clones presented had the vast majority of cells with same number of copies of chromosome 1 (four per cell) and chromosome 8 (two per cell). As such, we considered five copies of 1q12h a gain and three copies of 8c a gain in these populations. However, we did not verify that the clones had similar numbers of all other chromosomes.

### Data processing for TCGA breast cancer and lung adenocarcinoma

Complete methods for analysis of TCGA data are in the Supplemental Material.

### Competing interest statement

The authors declare the following competing financial interests: J.R.W. is a consultant for QSonica. J.K.J. is a consultant for Horizon Discovery. J.K.J. has financial interests in Editas Medicine, Hera Testing Laboratories, Poseida Therapeutics, and Transposagen Biopharmaceuticals. J.K.J.’s interests were reviewed and are managed by Massachusetts General Hospital and Partners HealthCare in accordance with their conflict of interest policies.

## Supplementary Material

Supplemental Material
