# Supplementary material for: Hypoxia drives transient site-specific copy gain and drug-resistant gene expression
Source: Genes Dev. 2015 May 15;29(10):1018–31. doi: 10.1101/gad.259796.115 (PMC4441050; doi:10.1101/gad.259796.115)
Supplement: Supplemental Material [file supp_29_10_1018__index.html]

Supplemental Material 

# Hypoxia drives transient site-specific copy gain and drug-resistant gene expression

## Supplemental Material

**Files in this Data Supplement:**

- Supp Fig S1.tif
- Supp Fig S2.tif
- Supp Fig S3.tif
- Supp Fig S4.tif
- Supp Fig S5.tif
- Supp Material.docx
- Supp Table S1.xlsx
- Supp Table S2.xlsx
- Supp Table S3.xlsx
