# Supplementary figures and images for: Hypoxia drives transient site-specific copy gain and drug-resistant gene expression
Source: Genes Dev. 2015 May 15;29(10):1018–31. doi: 10.1101/gad.259796.115 (PMC4441050; doi:10.1101/gad.259796.115)

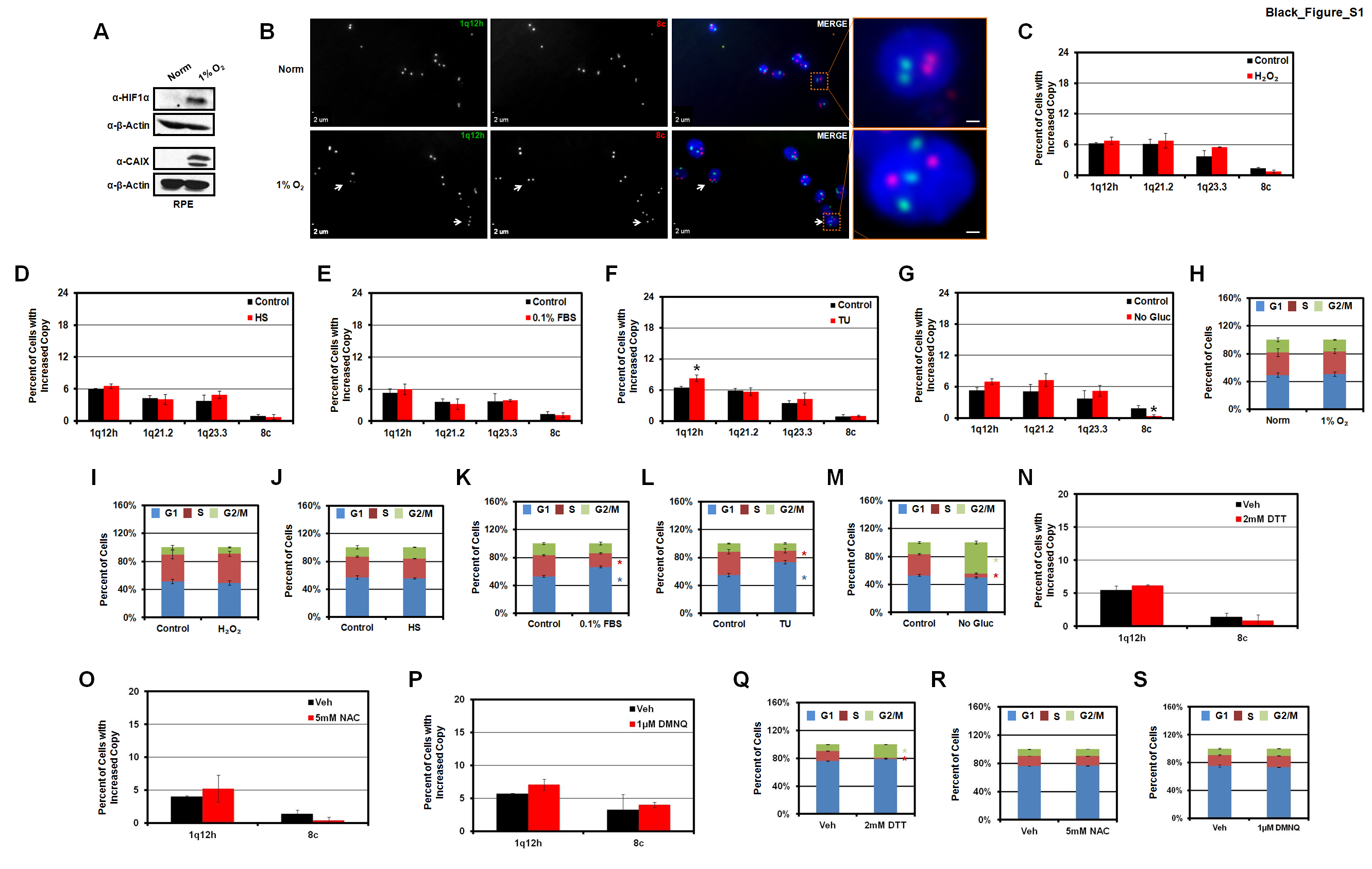

Supplement: Supplemental Material [file supp_29.10.1018_Black_Figure_S1.tif]

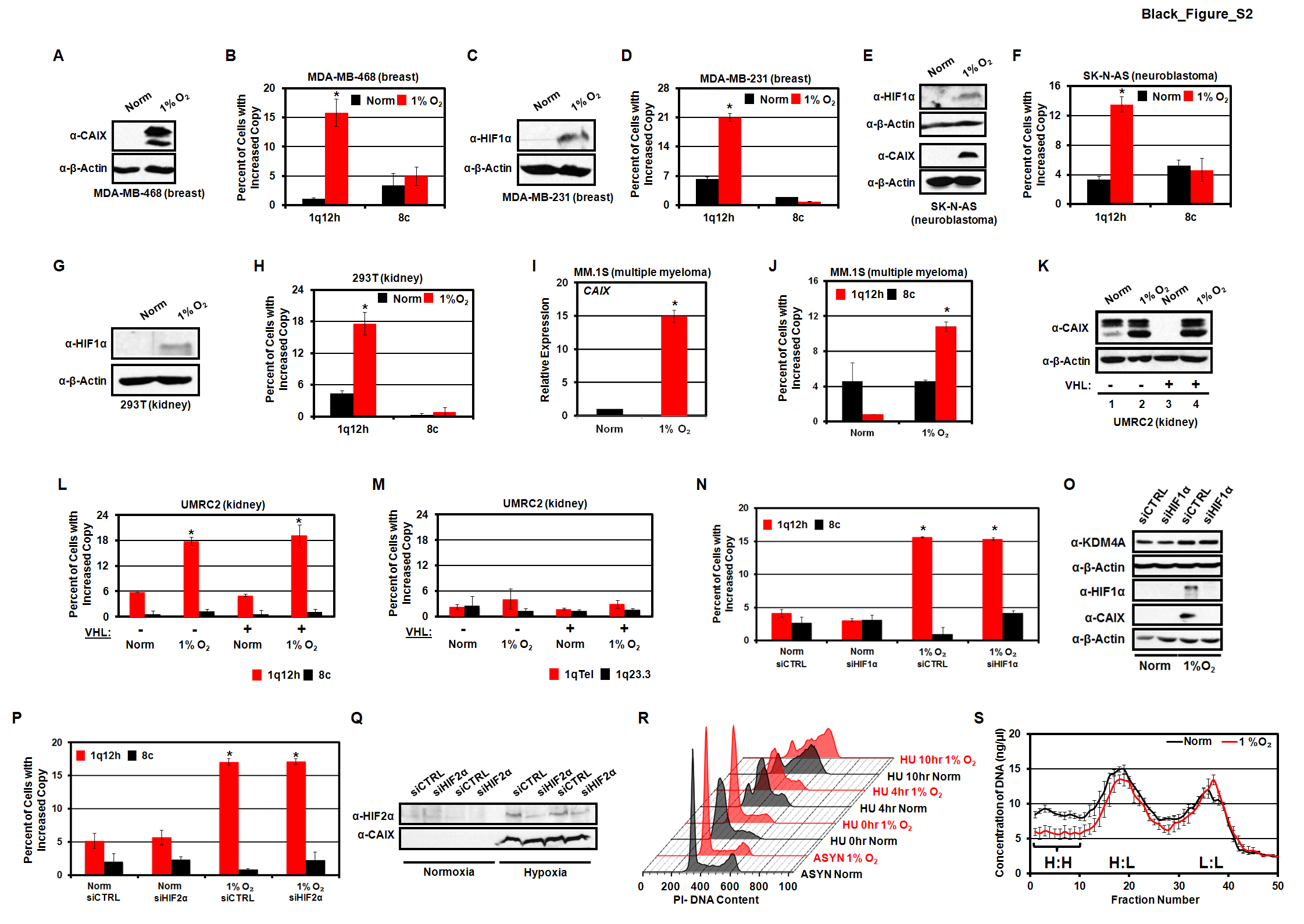

Supplement: Supplemental Material [file supp_29.10.1018_Black_Figure_S2.tif]

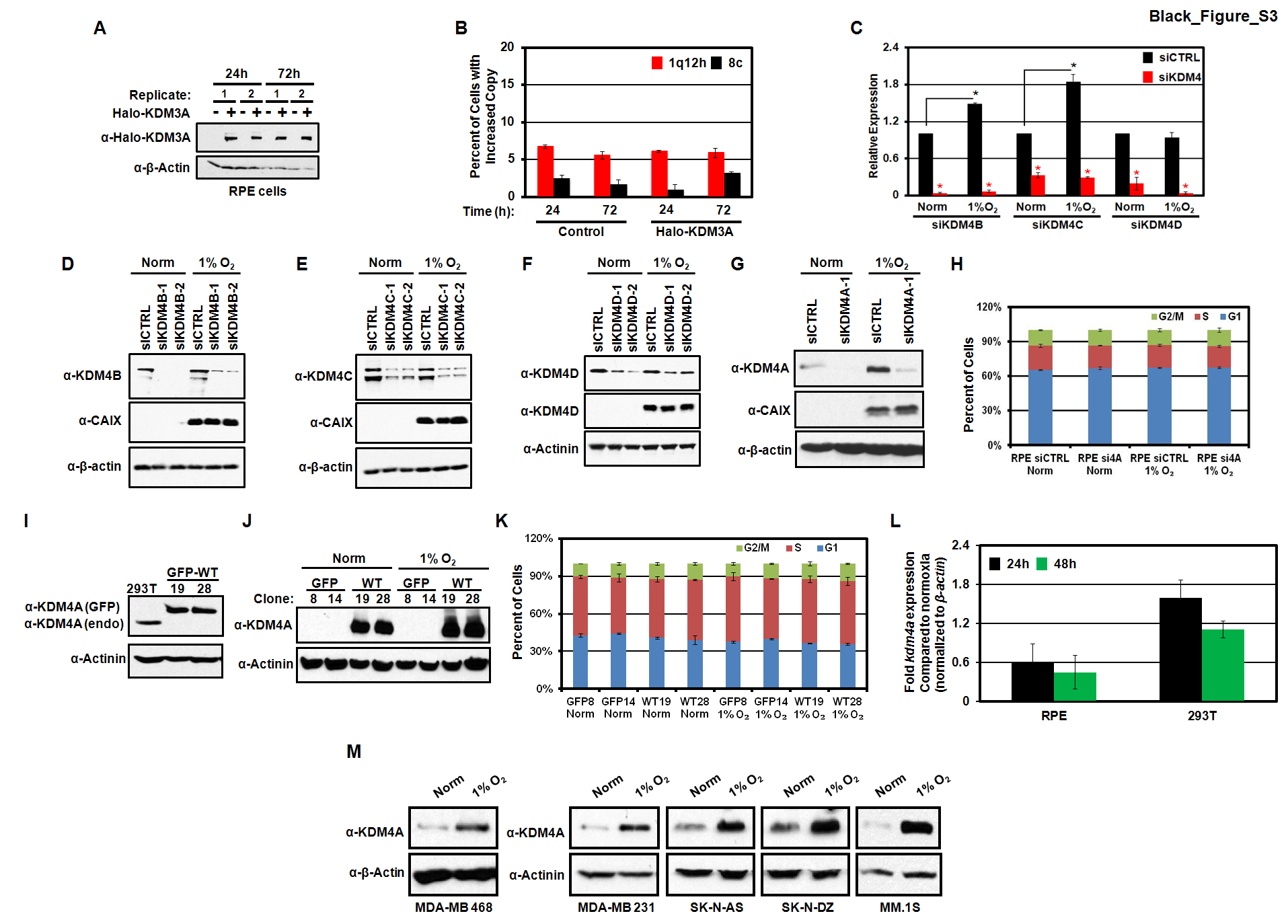

Supplement: Supplemental Material [file supp_29.10.1018_Black_Figure_S3.tif]

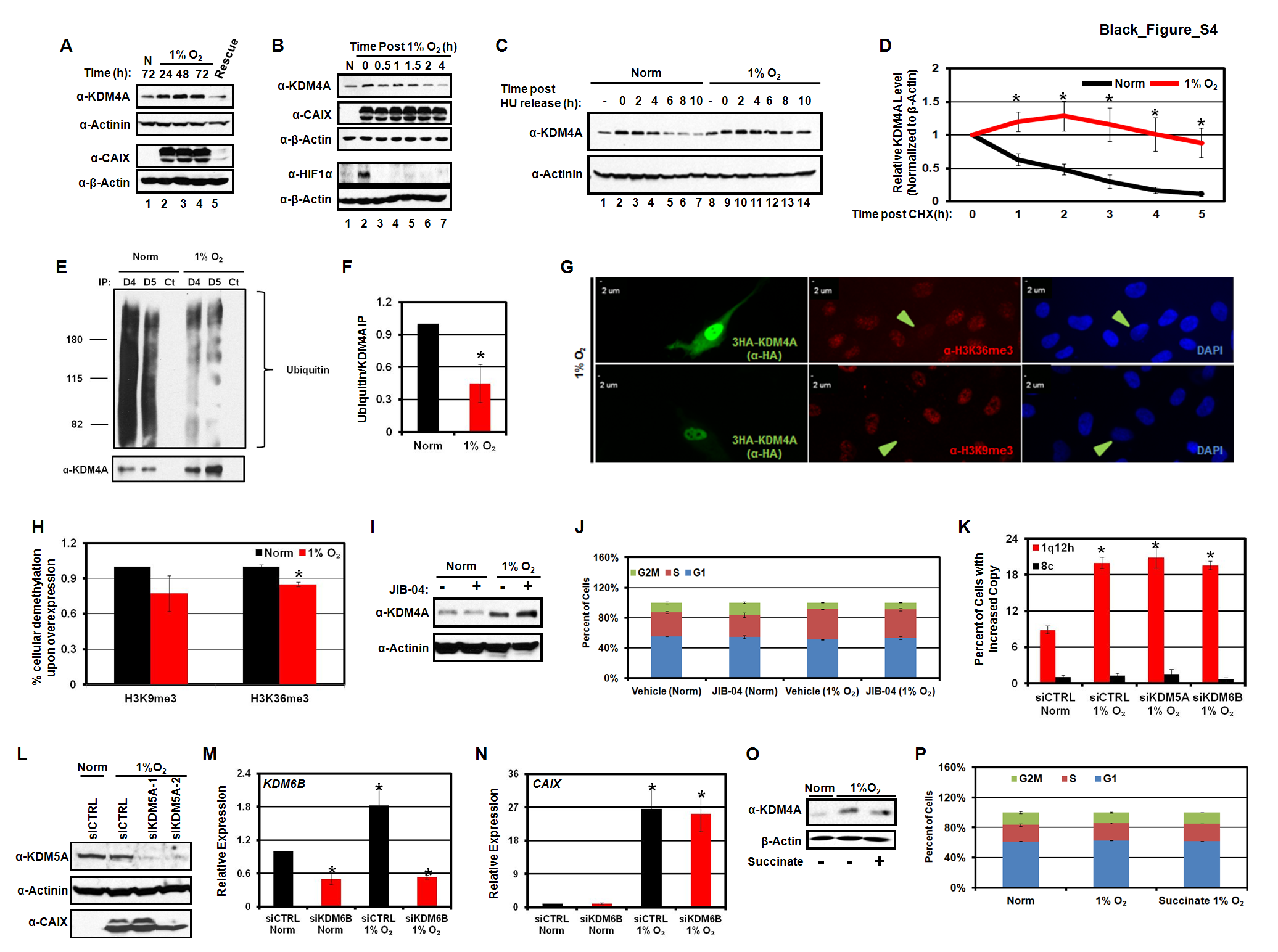

Supplement: Supplemental Material [file supp_29.10.1018_Black_Figure_S4.tif]

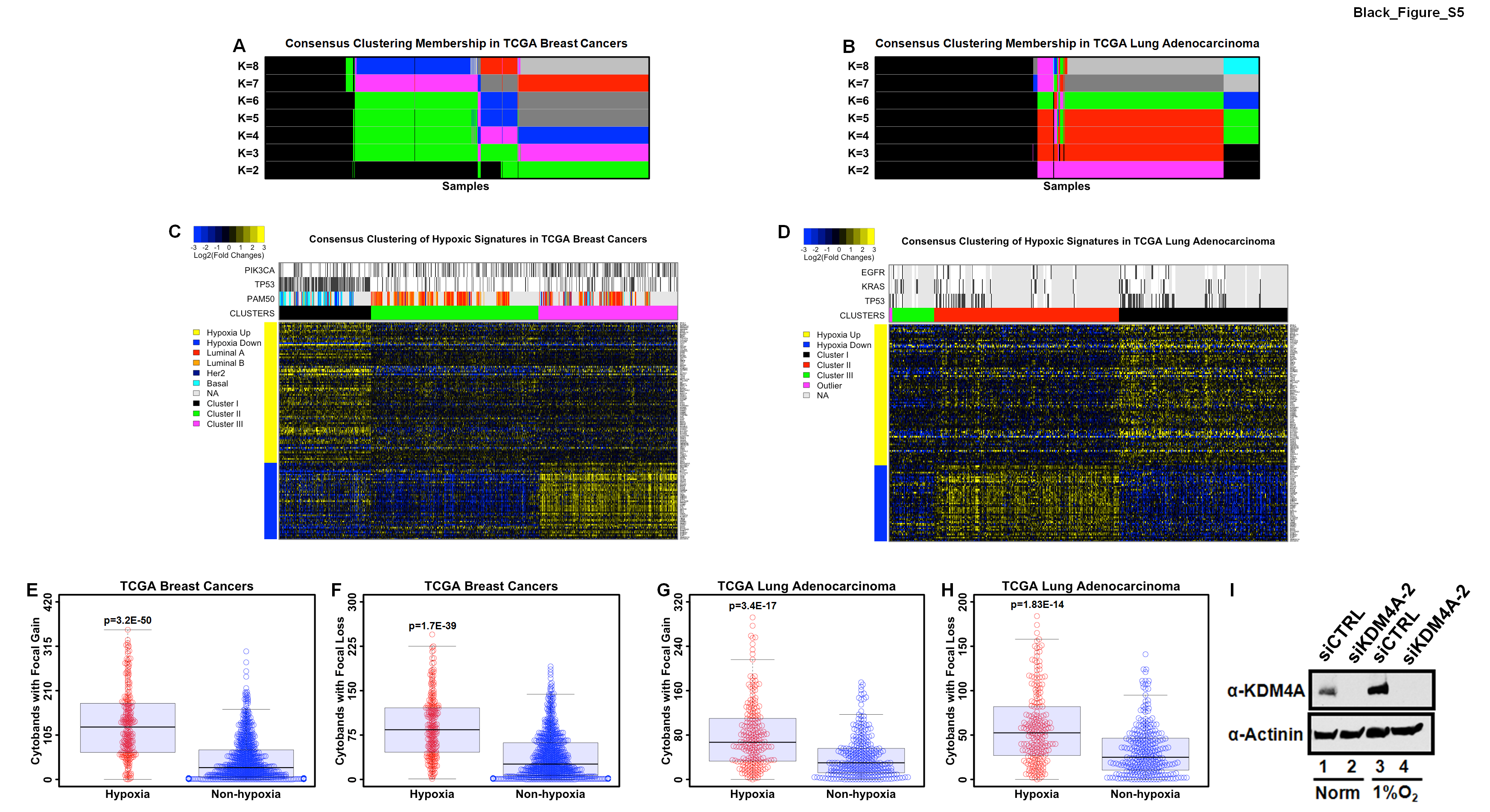

Supplement: Supplemental Material [file supp_29.10.1018_Black_Figure_S5.tif]
