# Supplementary material for: Hypoxia drives transient site-specific copy gain and drug-resistant gene expression
Source: Genes Dev. 2015 May 15;29(10):1018–31. doi: 10.1101/gad.259796.115 (PMC4441050; doi:10.1101/gad.259796.115)
Supplement: Supplemental Material [file supp_29.10.1018_Supp_Material.docx]

**Title: Hypoxia drives transient site-specific copy gain and drug resistant gene expression**

**Authors:** Joshua C. Black^1^†, Elnaz Atabakhsh^1^†, Jaegil Kim^2^†, Kelly M. Biette^1^, Capucine Van Rechem^1^, Brendon Ladd^1^, Paul d. Burrowes^1^, Carlos Donado^1^, Hamid Mattoo^1^, Benjamin P. Kleinstiver^3,4^, Bing Song^1^, Grasiella Andriani^5^, J. Keith Joung^3,4^, Othon Iliopoulos^1,6^, Cristina Montagna^5^, Shiv Pillai^1^, Gad Getz^2,3^, Johnathan R. Whetstine^1^*

# Affiliations:

# ^1^ Massachusetts General Hospital Cancer Center and Department of Medicine, Harvard Medical School, 13^th^ Street, Charlestown, Massachusetts 02129

^2^ Broad Institute of MIT and Harvard, Cambridge, Massachusetts 02142

^3^ Massachusetts General Hospital Cancer Center and Department of Pathology, Harvard Medical School, 13^th^ Street, Charlestown, Massachusetts 02129

^4^ Massachusetts General Hospital Center for Computational and Integrative Biology, 13^th^ Street, Charlestown, Massachusetts 02129

^5^ Department of Genetics, Pathology, Albert Einstein College of Medicine, Yeshiva University, Bronx, NY 10461

^6^ Massachusetts General Hospital Division of Hematology-Oncology, Department of Medicine, Boston, Massachusetts 02114

**†These authors contributed equally to this work**

# Running Title: Hypoxia generates site-specific copy gain

*Correspondence should be addressed to:

**Johnathan R. Whetstine**

Massachusetts General Hospital Cancer Center

Department of Medicine

Harvard Medical School

Building 149, Room 7-213

13^th^ Street, Charlestown, MA 02129

Tel: 617-643-4347, Fax: 617-724-9648

email: [jwhetstine@hms.harvard.edu](mailto:jwhetstine@hms.harvard.edu)

SUPPLEMENTAL MATERIAL:

Materials and Methods

Supplemental Figures S1-5

Supplemental Tables S1-3

**MATERIALS AND METHODS**

**Cell Culture and Transfections**

HEK293T (called 293T throughout), hTERT-RPE-1 (called RPE throughout), MDA-MB 231, MDA-MB 468, and UMRC2 cells were maintained in DMEM with 10% fetal bovine serum, 1% penicillin/streptomycin, and L-glutamine. SK-N-AS cells were maintained in DMEM/F12 (GIBCO) with 10% fetal bovine serum, 1% penicillin/streptomycin, and L-glutamine. MM.1S cells were maintained in suspension in RPMI with 10% fetal bovine serum, 1% penicillin/streptomycin, and L-glutamine. Zebrafish AB.9 cells ([Paw and Zon 1999](#_ENREF_9)) were purchased from ATCC and maintained in DMEM with 20% fetal bovine serum, 1% penicillin/streptomycin, and L-glutamine at 28˚C. Transient transfection experiments were performed using Roche X-tremeGENE 9 or Lipofectamine 3000 transfection reagent in OPTI-MEM I media (Gibco) for four hours or overnight. No selection was used in transient transfection experiments. siRNA transfections were carried out using Roche X-tremeGENE 9 siRNA reagent or Lipofectamine 3000 in OPTI-MEM I for four hours or overnight. Each siRNA experiment represents the average of at least two different siRNAs for each target gene.

**Hypoxic Conditions**

Cells were plated onto culture dishes and allowed to adhere for 20-24 hours in normoxia (5% CO_2_, 21% O_2_, and 74% N_2_). For hypoxic treatment, cells were maintained in a HERA Cell 150 incubator (Thermo Scientific) flushed with 5% CO_2_, 1% O_2_, and balanced with N_2_ for the duration of the experiment. Incubator calibrations and verifications were carried out by Bianchi Associates Calibrations/Verifications.

**Drug Treatments and Synchronization**

Cells were treated with the following chemical and metabolic stresses for 24 hours at doses used in the literature: 2µg/ml Tunicamycin (TU, Abcam), 60µM H_2_O_2_ (Thermo Fisher Scientific), reduced-serum DMEM (0.1% FBS), Glucose-free DMEM (No Gluc, GIBCO), 2mM DTT (Sigma), 5mM N-acetylcysteine (NAC, Sigma), and 1µM 2,3-Dimethoxy-1,4-naphthoquinone (DMNQ, Sigma). For heat shock (HS) treatment, cells were incubated at 43ºC for 30 minutes and returned to 37ºC for 24 hours prior to collection.

For G1/S synchronization, cells were treated with 2mM hydroxyurea (HU, Sigma) for 20 hours. To release, cells were washed twice with culture medium pre-conditioned in normoxia or hypoxia, and supplied with fresh pre-conditioned media. For JIB-04 treatment, normoxic cells were pre-treated with 62.5nM JIB-04 (Xcessbio) for 24 hours, and then treated again with JIB-04 and either transferred to 1% O_2_ or maintained in normoxia for an additional 24 hours. Succinate (Sigma, S9637) was administered at a final concentration of 2mM and cells were either maintained in normoxia for 72 hours or maintained in normoxia for 48 hours prior to being transferred to 1% O_2_ for 24 hours.

**Fluorescent In Situ Hybridization (FISH)**

FISH was performed as described in ([Manning et al. 2010](#_ENREF_6); [Black et al. 2013](#_ENREF_2)). Probes for 1q12h, 1q telomere, chromosome 8 centromere (alpha satellite), and X centromere (alpha satellite) were purchased from Rainbow Scientific. Probes for Zebrafish *BCL9* (CH73-15J19) and Zebrafish *IGBP1* (CH73-223D24) were purchased as BAC clones from Children’s Hospital Oakland Research Institute (CHORI BacPac) clone repository. Probes for 1q21.2 (*BCL9*) and 1q23.3 were purchased from Agilent (SureFISH). BACS were prepared utilizing PureLink HiPure Plasmid Filter Maxiprep kit (Life Technologies) using the recommended modified wash buffer. Probes were nick translated (Abbot Molecular Kit) in the presence of fluorescently labeled dTTP (Enzo Life Science). Images of multiple planes of fields of nuclei were acquired on an Olympus IX81 Spinning Disk Microscope and analyzed using Slidebook 5.0 software. We used a conservative scoring metric for copy gain. Any foci that were touching were scored as a single copy to prevent increased numbers due to normally replicated foci. For RPE cells, copy gain was scored as any cell with 3 or more distinct foci. For 293T cells, copy gain was scored for any cell with 5 or more distinct foci. For UMRC2 cells, copy gain was scored for any cell with 6 or more foci. For SK-N-AS cells, copy gain was scored for any cell with 5 or more foci. For MDA-MB-468 cells, copy gain was scored for any cell with 5 or more foci. For MDA-MB-231 cells, copy gain was scored for any cell with 7 or more foci. Approximately 100 cells for each replicate were scored for all experiments. All FISH experiments include at least 2 biological replicates. For each experiment, at least one replicate includes FACS and western blot from the same cells used for FISH. For knockdown experiments, at least two different siRNA were used for each target. Results are presented as the average from both of the independent siRNAs.

**Antibodies**

Antibodies used were: KDM4A (Neuro mAB, 75-189), KDM4B (Santa Cruz, sc-67192), KDM4C (Abcam, ab85454), KDM4D (Abcam, ab93694), KDM5A (Abcam, ab70892), β-actin (Millipore), RFP (Abcam, ab62341), Halo (Promega), Actinin (Santa Cruz, sc-17829), HA 12CA5 (Roche), HIF1α (Santa Cruz, sc-10790), HIF2α (Cell Signaling, Clone D9E3), CAIX (Abcam, ab108351), LDH1 (Santa Cruz, sc-133123), Histone H3 (Abcam, ab1791), HA.11 (Covance), KDM4A-P006 FAB (SGC), FBXL4 (Santa Cruz, sc-54489), FBXW2 (abcam ab83467), Cul1 (Santa Cruz, sc-17775), Ubiquitin (Santa Cruz, sc-8017).

**Western Blots**

Western blots were performed as in ([Black et al. 2010](#_ENREF_1)). Briefly, adherent cells were either scraped directly into PBS, or washed with PBS, trypsinized and collected by centrifuging at 2,000 RPM for 5 minutes. For preparation of whole-cell lysates, cell pellets were washed once in ice-cold PBS and resuspended in RIPA lysis buffer [50mM Tris pH 7.4, 150mM NaCl, 0.25% Sodium Deoxycholate, 1% NP40, 1mM EDTA, 10% Glycerol] supplemented with cOmplete protease inhibitor and PhosSTOP phosphatase inhibitor cocktails (Roche). Cells were lysed on ice for 15 minutes and immediately frozen at -80ºC for 10 minutes. Lysates were subsequently sonicated at 70% amplitude for 15 minutes in a QSonica Q700 sonicator and cleared of cell debris by centrifuging at 12,000RPM for 15 minutes, before being analyzed by western blotting. For HIF1α and HIF2α expression, adherent cells were washed twice with ice-cold PBS and scraped directly in warmed 1x Laemmli buffer. Samples were sonicated at 70% amplitude for 15 minutes in a QSonica Q700 and boiled at 95 ºC for 10 minutes immediately prior to western blotting.

**Expression Plasmids and siRNAs**

pCS2-3HA-huKDM4A and pCS2-3HA-zfKDM4A WT and catalytic mutants were prepared by gateway transfer into pCS2-3HA. All clones were sequence verified. Silencer Select siRNAs were purchased from Life Technologies, as follows: KDM4A (s18636, s18637, s18635), KDM4B (s22867, s229325), KDM4C (s22989, s225929), KDM4D (s31266, s31267), KDM5A (s11834, S11836), KDM6B (s23109, s23110), HIF1α (s6539, s6541), HIF2α (s4698, s4700). Results for FISH with each siRNA (at least 2 independent siRNA per target) were averaged together in all knockdown experiments presented.

**RNA Extraction and Quantitative PCR**

Cells for RNA isolation were collected by scraping or trypsinization and washed twice with PBS. Cells were resuspended in Tri-Reagent (Roche) and stored at -80°C until use. RNA was isolated using the miRNAeasy Plus kit with on-column DNAse digestion (Qiagen) following the manufacturer’s instructions and quantified using a Nanodrop 1000D. Single strand cDNA was prepared using the Transcriptor First Strand cDNA Synthesis Kit (Roche) with oligo dT primers. Expression levels were analyzed by quantitative real time PCR in a Lightcycler 480 with FastStart Universal SYBR Green Master (Roche) following the manufacturers protocols. All samples were normalized by comparison to β-actin transcript and hypoxia induction was verified with primers for CAIX. For CKS1B transcript analysis, we observed transcript induction in hypoxia in all samples from untreated MDA-MB-231 cells (Fig. 6A). However, transfection of MDA-MB-231 cells reduced the induction level of CKS1B (we considered >1.15-fold induced, Fig. 6D) and resulted in induction in 16 of 24 replicates, siKDM4A depletion resulted in reduced CKS1B transcript in 15 of 16 induced replicates. Replicates included three different KDM4A siRNA. The data represent an average of all replicates that exhibited induction of CKS1B in hypoxia (16 of 24). *CKS1B* was amplified (FISH) in all replicates and not amplified upon KDM4A depletion. Primers available upon request.

**Catalytic Activity of huKDM4A and zfKDM4A in Hypoxia**

Assays for demethylase activity were performed using immunofluorescence as described in ([Whetstine et al. 2006](#_ENREF_13)). Briefly, The indicated HA-tagged KDM4A constructs were transfected into RPE cells grown on coverslips in 6-well dishes using X-tremeGENE 9 (Roche) or Lipofectamine 3000 (Life Technologies) DNA transfection reagent. Following 24 or 48 hours in hypoxia, H3K36me3 and H3K9me3 were assayed by examining transfected cells (positive for HA staining; HA.11 Covance) following fixation ([Whetstine et al. 2006](#_ENREF_13); [Black et al. 2013](#_ENREF_2)). Approximately fifty highly transfected cells in each of two biological replicates were scored for each condition. Data presented for normoxia is an average of the two replicates. For hypoxia, data are presented as the percent of activity of the same construct under normoxic conditions for each of two biological replicates, which were averaged together.

**Human CD4+ T cell purification and *in vitro* culture**

Buffy coats (Sanguine Biosciences) or peripheral blood of healthy controls was diluted 1:2 in room-temperature PBS lacking Ca^2+^/Mg^2+^. Mononuclear cells were isolated by Ficoll-Paque Plus (GE Healthcare) density-gradient centrifugation following the manufacturer's protocol. PBMCs were resuspended at a density of 20x10^6^ cells/mL and reacted with Fc receptor blocking solution (Human TruStain FcX, Biolegend), followed by surface staining with APC anti-human CD4 antibody (Clone OKT4, Biolegend) for 45 minutes on ice. Antibody-stained cells were resuspended in HBSS (GIBCO) supplemented with 10mM glucose and sorted by flow cytometry. Sorted cells (including CD4+ T cells) were collected in 5mL tubes containing 1mL collection medium (DMEM supplemented with 30% FBS) and reanalyzed by flow cytometry to ensure ≥99% purity in defined gates. Sorted cells were allowed to recover in RPMI medium (GIBCO) supplemented with 10% FBS for 2 hours. For resting CD4+ T cell culture, cells were seeded onto 60mm dishes and maintained in complete medium supplemented with 10ng/mL recombinant human interleukin-2 (rhIL-2, R&D Systems). For stimulated CD4+ T cell culture, 60mm dishes were pre-coated with a cocktail containing 5µg/mL anti-human CD3 (Clone HIT3a, Biolegend) and 3µg/mL anti-human CD28 (Clone CD28.2, Biolegend) for 1 hour, after which cells were seeded onto the coated dish. Stimulated CD4+ T cells were maintained in complete medium supplemented with 10ng/mL rhIL-2, and anti-CD3/CD28 antibodies. Resting and stimulated CD4+ T cells were allowed to recover for 24 hours in normoxia (21% O_2_), followed by an additional 24 hours in normoxia or in hypoxia (1% O_2_) prior to being collected.

**Half-Life Determination**

Protein turnover was assessed as outlined in ([Van Rechem et al. 2011](#_ENREF_11)). Briefly, cells maintained in normoxia and hypoxia were treated with 400µM Cycloheximide (Sigma) for the indicated time, after which lysates were prepared and analyzed by western blot.

**Immunoprecipitation**

Immunoprecipitations were carried out as in ([Van Rechem et al. 2011](#_ENREF_11)) on cells grown in normoxia or hypoxia for 24 hours. KDM4A was immunoprecipitated from whole-cell lysates using KDM4A-P006, KDM4A-P014, and KDM4A rabbit polyclonal antibody ([Black et al. 2010](#_ENREF_1); [Van Rechem et al. 2015](#_ENREF_12)). For ubiquitination determination, KDM4A IPs were washed under denaturing conditions as in ([Van Rechem et al. 2011](#_ENREF_11)). Ubiquitination of KDM4A was quantitated using ImageJ and normalized to the amount of KDM4A IP’d.

**Cesium Chloride Gradient Centrifugation**

CsCl density gradient centrifugation was performed as in ([Black et al. 2013](#_ENREF_2)). Briefly, RPE cells were grown in normoxia or 1% O_2_ for 24 hours prior to addition of BrdU. Cells were labeled with BrdU for 12 hours and 45 minutes. Each rereplicated fraction was diluted to 15ng/ul stock and 7.5ng of rereplicated DNA pool was analyzed by qPCR on a Roche LC480 using FastStart Universal SYBR Green Master Mix (Roche) following the manufacturer’s instructions. 7.5ng of input DNA was analyzed by qPCR at the same time. Each sample was normalized to its own input prior to determination of fold-change in rereplication. Primers used in this study will be provided upon request.

**Flow Cytometry and Cell Cycle Analysis**

Asynchronously growing, or G1/S arrested cells were prepared and fixed as in ([Black et al. 2010](#_ENREF_1)). Cells were stained with 10µM EdU for 1 hour prior to collection. Cell cycle was analyzed by PI staining or EdU incorporation using Click-IT EdU Flow Cytometry Assay Kit (Life Technologies). Flow cytometry of CD4+ T cells and cell cycle distribution were analyzed using a BD FACS ARIA II.

**Cell Fractionation**

Cytoplasmic, nuclear and chromatin fractions were prepared from RPE cells. Cell pellets were washed twice in ice cold PBS and resuspended in ice cold Buffer A (10mM HEPES pH 7.9, 10mM KCl, 0.1M EDTA, 0.5M EGTA) and incubated on ice for 15 minutes. Swollen cells were lysed by addition of NP-40 to 0.8% with 10 seconds of vortexing. Lysed cells were centrifuged and the supernatant kept as cytoplasm. The nuclear pellet was resuspended in Buffer C (10mM HEPES pH 7.9, 400mM NaCl, 1mM EDTA, 5mM EGTA), dounced to resuspend the nuclei and incubated at 4˚C for 30 minutes with rotation. Extracts were centrifuged and the supernatant kept as nuclear extract. Chromatin pellets were resuspended in N-Buffer (20mM Trish pH 7.5, 100mM KCl, 2mM MgCl2, 1mM CaCl2, 0.3M Sucrose, 0.1% Triton X-100, 3U per ml micrococcal nuclease). Samples were sonicated for 10 minutes at 70% amplitude in a Q700 cup horn (QSonica) and then incubated at room temperature for 15 minutes for MNase digestion. Reactions were stopped by addition of 5mM EGTA and centrifuged to clear. Supernatant was kept as chromatin extract.

**Spectral Karyotyping**

SKY and corresponding analysis was performed by Cristina Montagna in the Molecular Cytogenetic Core at Albert Einstein College of Medicine. Two biological replicates of cells grown in normoxia or hypoxia for 24 hours were analyzed by SKY.

**Generation of KDM4A knockout 293T cells using CRISPR/Cas9**

A *KDM4A*-targeting CRISPR guide RNA (gRNA) was designed using the ZiFiT Targeter web server as previously described ([Fu et al. 2014](#_ENREF_4)). This guide sequence targeted (CTTTACTCAGTACAACATAC) at position 243-262 in *KDM4A* cDNA. The gRNA was cloned into the BsmBI-digested expression plasmid pMLM3636 as described ([Fu et al. 2014](#_ENREF_4)).

For generation of *KDM4A* knockout CRISPR cell lines, 293T cells were seeded onto 24-well dishes and transfected with the Cas9 nuclease (pJDS246) and gRNA using Lipofectamine 3000 ([Fu et al. 2013](#_ENREF_3)). Forty-eight hours post-transfection, cells were collected and plated as single cells in 96-well dishes. Twenty-eight days post-seeding, genomic DNA and whole cell lysates were collected and clones exhibiting mutations in *KDM4A* were identified using T7E1 assays and western blotting ([Fu et al. 2014](#_ENREF_4)). Homozygous deletion of *KDM4A* in the selected cell line was further validated by sequencing of genomic loci.

We generated genetic rescue lines by reintroducing GFP or GFP-KDM4A. *KDM4A* deficient cell lines expressing either GFP or GFP-KDM4A were generated using retroviral infections of pMSCV-GFP or pMSCV-GFP-KDM4A as described in ([Black et al. 2013](#_ENREF_2)). GFP-positive cells were isolated by cell sorting on a FACS ARIA II. Following recovery, GFP and GFP-KDM4A cells were replated as single cells. Independently derived, single-cell clonal lines were established. Expression of GFP or GFP-KDM4A, was confirmed by western blot and no detectable endogenous KDM4A was observed. As clones were derived from 293T cells, clonal variability for chromosome numbers was observed (*i.e.* chromosome 1). The independent clones presented had the vast majority of cells with same number of copies of chromosome 1 (four per cell) and chromosome 8 (2 per cell). As such, we considered 5 copies of 1q12h a gain and 3 copies of 8c a gain in these populations. However, we did not verify that the clones had similar numbers of all other chromosomes.

**Data Processing for TCGA Breast Cancer and Lung Adenocarcinoma**

All genomic data of mutation, copy number, and mRNA expression for TCGA Breast Cancer (BRCA) and Lung Adenocarcinoma (LUAD) were downloaded from Broad GDAC (Genome Data Analysis Center) Firehose analysis run named “15 January 2014” (doi:10.7909/C1H41PXV). Complete methods for all computational analysis can be found in Supplemental Experimental Procedures.

Copy Number Data: The segmented copy number data for 1007 BRCA samples and 493 LUAD samples was processed by GISITC2.0 ([Mermel et al. 2011](#_ENREF_7)) to annotate the somatic copy number alterations (SCNAs) for 24,174 genes. Copy-number data were dissociated to arm-level and focal copy-number alterations as described in the GISTIC2.0 paper ([Mermel et al. 2011](#_ENREF_7)). In addition to the copy number annotation for each gene, the mean focal copy number for 807 cytobands including X chromosome were calculated for each sample by taking the average of the focal SCNA values across all genes within a cytoband. The contribution of arm-level SCNAs to the mean cytoband focal copy was eliminated by only considering GISTIC annotated focal copy numbers spanning a much smaller region than a chromosome arm.

RNA-seq Data: The mRNA expression levels for 18264 genes in 1019 BRCA samples and 488 LUAD samples were annotated by the log_2_-normalized RSEM (RNASeq by Expectation Maximization ([Li and Dewey 2011](#_ENREF_5))) values. RSEM values for 956 BRCA samples and 486 LUAD samples having copy number data were median-centered (by subtracting the median expression across tumor samples), yielding log_2_ (Fold Changes) and utilized in the downstream analysis.

Somatic Mutation Data: The MAF (Mutation Annotation Format) file for 976 BRCA samples and 229 LUAD samples contained 73,729 and 92,133 somatic mutations, respectively.

BRCA subtype information: The subtype information for 504 BRCA samples based on PAM50 gene set was extracted from the supplemental data (BRCA.547.PAM50.SigClust.Subtypes.txt) of TCGA BRCA paper ([Network 2012](#_ENREF_8)).

**Hypoxia Signature Gene Set**

The hypoxia metagene ([Winter et al. 2007](#_ENREF_15)), was downloaded from MSigDB ([Subramanian et al. 2005](#_ENREF_10)) and used as a hypoxia signature gene set in a downstream analysis. The efficacy of this gene set was demonstrated as a significant prognostic factor for overall survivals in both HNSC and BRCA data set. The final hypoxia signature gene set (Supplemental Table S3) was comprised of 92 up-regulated (HS-up) and 52 down-regulated (HS-down) genes including well-known hypoxia biomarkers such as HIF1A, CA9, and VEGFA.

**Identifying Hypoxia Samples using Consensus Hierarchical Clustering**

We utilized consensus hierarchical clustering to identify a cluster of samples that showed the most concordant expression pattern to the previously-defined hypoxia signature gene set ([Winter et al. 2007](#_ENREF_15)). Using the mRNA expression data, we first computed the Spearman correlation coefficients between pairs of samples using the median-centered log_2_-normalized RSEM values. We applied the consensus hierarchical clustering R package *ConsensusClusterPlus* ([Wilkerson and Hayes 2010](#_ENREF_14)), with 1-Spearman correlation as a distance metric, and run over 1000 iterations of the “average linkage” method and 80% resampling rate. We varied the number of clusters from K=2 to 8. We determined the hypoxia cluster by examining the stability of the chosen cluster throughout K and the concordance of mRNA expression levels in each cluster to the known expression patterns of hypoxia up or down signatures. This process finally resulted in the choice of K=3 in BRCA (Figs. S5A and S5C) and K=4 in LUAD (Figs. S5B and S5D). Details described below:

(1) TCGA Breast Cancers

The cluster membership of samples across K is illustrated in Fig. S5A, where the most hypoxia-related cluster at any given K (chosen based on the mean expression levels of the hypoxia-up genes) was highlighted in “black” and other colors represent different clusters. At K=2 almost 60% samples belonged to the hypoxia cluster (black). Half of these samples were separated from the large black cluster and formed their own cluster (green) at K=3. The samples in the black cluster (35%) at K=3 had the most concordant expression pattern to both the up and down genes in the signature (“hypoxia-signature concordant cluster”), while the green cluster (42%) had an overall down-regulations regardless of hypoxia signatures (“hypoxia-signature neutral cluster”). On the other hand, the magenta cluster at K=3 (23%) had an opposite expression pattern to the known hypoxia signature (“hypoxia-signature discordant cluster”), which is also observed in the expression heatmap in Fig. S5C. We also observed that most samples in the black cluster at K=3 consistently remained in the hypoxic cluster up to K=8, indicating the strong stability of this cluster throughout K. Interestingly, the Basal (65 out of 88) and Her2 (31 out of 55) breast cancer subtypes were significantly enriched in the hypoxia cluster, while most Luminal A/B (322 out of 341) and eight Normal-like samples were in the non-hypoxia cluster.

(2) TCGA Lung Adenocarcinoma

Both clustering results at K=2 and 3 had a very similar stratification of samples except for two outlier samples (magenta) in K=3 (Fig. S5B). Crossing from K=3 to 4 a small number of samples with a much weaker hypoxia-up signature were separated from the black hypoxia cluster, forming the green cluster at K=4. The majority of samples (42%) remained in the hypoxic cluster had the most concordant expression pattern to both up and down signatures (“hypoxia-signature concordant cluster”), while in the green cluster (11%) all hypoxia signature genes were down-regulated , hence called the “hypoxia-signature neutral cluster” (Fig. S5D). On the contrary, the red cluster at K=4 (46%) had a largely discordant expression pattern with respect to the hypoxia signature (called the “hypoxia-signature discordant cluster”). Ignoring four outlier samples (magenta) at K=4 the stratification of samples into hypoxia-concordant, neutral, and discordant groups is analogous to the partitioning of BRCA at K=3. Most samples in the black cluster at K=4 remained in the hypoxic cluster up to K=8, demonstrating the strong stability of the chosen hypoxia cluster.

**Time to Death versus Hypoxia**

A short follow-up time for survival (median survival time - 21.1 months in BRCA and 12.4 months in LUAD) and a high fraction of censored samples (820 out of 920 samples in BRCA and 313 out of 430 samples in LUAD) is a significant challenge in evaluating the association of hypoxia samples with clinical outcome. Instead, we examined the association with the number of deceased patients, illuminating a significant higher risk in the hypoxic samples in both BRCA (Fig. 5A, p< 0.00011) and LUAD (Fig. 5B, p<0.0097) by the one-tailed Wilcoxon rank-sum test.

**Chromosomal Instability vs Hypoxia**

In order to examine whether the hypoxia samples had a significant enrichment of the chromosomal instability, the distributions of the number of cytobands harboring focal gains (mean cytoband focal copy > 0) and/or losses (mean cytoband focal copy < 0) per sample were compared between hypoxia and non-hypoxia samples for BRCA (Figs. 5C, Fig. S5E,F) and for LUAD (Figs 5D, Fig. S5G,H).

**Detecting Chromosomal Regions Significantly Associated to Hypoxia**

Since the amplification of *KDM4A* can induce a site-specific copy gain at 1q21 ([Black et al. 2013](#_ENREF_2)), samples with a focal copy gain of the 1p34.1 cytoband (where *KDM4A* resides) were excluded from downstream analysis (76 samples in BRCA and 46 samples in LUAD). Detecting chromosomal regions (*i.e.,* cytobands) significantly associated with hypoxic samples was performed by the statistical test based on the normal approximation for the null distribution of mean cytoband copy difference between hypoxia and non-hypoxia samples. The null distribution was approximated by a normal density function with the population mean difference, m1 – m0, and the variances of S1/n1 + S0/n0. Here m1 and m0 are sample means, S1 and S0 are sample variances, and n1 and n0 are the number of samples in the hypoxia and the non-hypoxia group. The p-values for mean cytoband copy gains in hypoxia samples were computed by computing the probability of more extreme differences than the observed copy difference in the null distribution across 807 cytobands.

**SUPPLEMENTARY FIGURE LEGENDS**

**Fig. S1. Treatment with chemical and metabolic stresses does not promote copy gain.** (A) Hypoxic conditions increase HIF1α and CAIX levels in RPE cells. Western blot indicating protein levels of HIF1α and CAIX in normoxia or following 24 hours in hypoxia (1% O_2_). (B) Hypoxia induces copy gain of 1q12h. Representative images demonstrating copy number of 1q12h and 8c in RPE cells maintained in normoxia or hypoxia. Arrows indicate cells with three copies of 1q12h in hypoxia. The dashed boxes identify nuclei presented at higher magnification at the right of the panel. (C-G) Treatment with chemical and metabolic stresses does not promote copy gain. Quantification of FISH for 1q12h, Chr 8, 1q23.3 and 1q21.2 after 24 hours of ROS (H_2_O_2_) (C), 43ºC heat shock (HS) (D), reduced serum (0.1% FBS) (E), Tunicamycin (TU) (F), and glucose deprivation (G). (H-M) Cell cycle analysis following 24 hours exposure to the indicated stresses. (N-S) Oxidants and reducing reagents do not induce site-specific copy gains. Quantification of FISH for 1q12h and 8c (N-P) and cell cycle analysis (Q-S) in RPE cells following 24 hours of treatment with 2mM DTT, 5mM N-acetyl Cysteine (NAC), and 1µM DMNQ. In all panels, error bars represent the S.E.M. * indicates significant difference from control samples by two-tailed Student’s t-test (p<0.05). * adjacent to bar graphs for cell cycle distribution indicate p<0.05 compared to control samples for that cell cycle phase (Blue=G1, Red= S, Green= G2/M).

**Fig. S2. Hypoxia promotes site-specific copy gains in diverse cancer cell types.**  (A-D) Hypoxia promotes site-specific gains in breast cancer cell lines. Western blots depict the hypoxic response of MDA-MB 468 (A) and MDA-MB 231 (C) cells following 24 hours of hypoxic exposure. Quantification of FISH indicates amplification of 1q12h but not 8c in hypoxic MDA-MB 468 (B) and MDA-MB 231 (D) cells. (E-J) SK-N-AS neuroblastoma (E,F), 293T kidney (G,H), and MM.1S multiple myeloma (I,J) cells are hypoxic and exhibit copy gain of 1q12h following 24 hours of 1% O_2_. (K-M) Hypoxia promotes site-specific gain in renal cancer cells independent of activated HIF1/2α (UMRC2 – lack *VHL* and have constitutively active HIF). (K) Western blot indicating the hypoxic response of UMRC2 cells lacking (-) or expressing (+) VHL following 24 hours in hypoxia. (L,M) Quantification of FISH for 1q12h and 8c (L) or 1q23.3 and 1qtel (M) after 24 hours of normoxia or 1% O_2_. (N) Hypoxia-induced copy gains are not dependent on HIF1α. Quantification of FISH for 1q12h and Chr 8 in RPE cells maintained in either in normoxia or 1% O_2_, with or without depletion of HIF1α. (O) Western blot demonstrating abrogation of CAIX induction upon HIF1α depletion. (P) Hypoxia-driven copy gains are not dependent on HIF2α. Quantification of FISH for 1q12h and Chr 8 in RPE cells maintained in either in normoxia or 1% O_2_, with or without depletion of HIF2α. (Q) Western blot demonstrating CAIX induction upon HIF2α depletion. (R) Representative FACS analysis demonstrating cell cycle progression through HU release in normoxia and hypoxia. Cell cycle profiles are provided for asynchronous (ASYN), HU arrested (0hr), and released (4hr and 10hr) cells at normoxia or 1% O_2_. (S) A graph of the CsCl density gradient profile from the normoxia and hypoxia triplicate samples used in the rereplication experiment. Positions of the light:light (L:L; no replication), heavy:light (H:L; normal replication) and heavy:heavy (H:H; rereplicated) are indicated. Error bars represent the S.E.M. * indicates significant difference from control samples by two-tailed Student’s t-test (p<0.05).

**Fig. S3.** **Hypoxia stabilizes KDM4A protein levels.** (A,B) Overexpression of KDM3A does not promote copy gain. Western blot depicting overexpression of Halo-KDM3A for 24 or 72 hours (A), which is insufficient to promote copy gain of 1q12h (B). (C-F) Depletion of KDM4B-C does not impede hypoxia-mediated copy gain. (C) siRNA-directed depletion of KDM4B,C or D in normoxic and hypoxic RPE cells was verified by qRT-PCR analyses. (D-F) Western blot confirming depletion of KDM4B (D), KDM4C (E) and KDM4D (F) in RPE cells maintained in normoxia and hypoxia. (G) Western blot depicting siRNA-mediated depletion of KDM4A under normoxic and hypoxic conditions. (H) Cell cycle profile following siRNA depletion of KDM4A in normoxia and hypoxia. (I-K) Genomic deletion of *KDM4A* using CRISPR/Cas9 abrogates KDM4A expression. (I) Western blot indicating relative KDM4A protein levels in 293T parental (293T) and 293T CRISPR cell lines expressing GFP-KDM4A (WT19 and WT28). ‘”Endo” indicates endogenous KDM4A in parental 293T cells, while “GFP” indicates exogenous GFP-KDM4A reintroduced in to WT19 and WT28. (J) A western blot demonstrating KDM4A protein levels in 293T CRISPR cell lines stably expressing GFP and GFP-KDM4A upon normoxic and hypoxic exposure. Lanes were spliced together from different regions of the same exposure of the same blot. (K) Cell cycle profiles of 293T CRISPR GFP and GFP-KDM4A cell lines in normoxia or hypoxia. (L) KDM4A transcript levels do not correlate with increased protein observed in hypoxia. KDM4A mRNA levels were analyzed by qRT-PCR and normalized to β-actin. (M) Hypoxia increases KDM4A protein levels in breast (MDA-MB-468 and MDA-MB-231), neuroblastoma (SK-NAS and SK-N-DZ), and myeloma (MM.1S) cell lines. For all panels, error bars represent the S.E.M. * indicates significant difference from control samples by two-tailed Student’s t-test (p<0.05).

**Fig. S4.** **KDM4A protein levels are dynamic and correlate with hypoxia treatment.** (A) KDM4A levels are increased in hypoxia but return to baseline when cells are returned to normoxia (Rescue). (B) KDM4A levels return to baseline within four hours of return to normoxia. KDM4A levels were analyzed by western blot at the indicated times after a 48 hour 1% O_2_ treatment. (C) Western blot depicting KDM4A levels in asynchronous (-) and HU arrested and released cells in hypoxic and normoxic conditions. (D) Hypoxia increases the half-life of KDM4A in 293T cells. Quantification of half-life indicates a half-life of 1hr 51min ± 28min in normoxia and 6hr 13min ± 10min in hypoxia. * indicates significant difference from control samples at the same time point by two-tailed Student’s t-test (p<0.05). (E,F) KDM4A ubiquitination is decreased in hypoxic conditions. (E) KDM4A was immunoprecipitated from 293T cells maintained in normoxia or hypoxia using KDM4A-P006 (D4) and KDM4A-P014 (D5) ([Van Rechem et al. 2015](#_ENREF_12)). IPs were washed under denaturing conditions and analyzed by western blotting. (F) Graphical representation of KDM4A ubiquitination in normoxia and hypoxia. Quantification of ubiquitination indicates an approximately 2.2-fold reduction in ubiquitination upon exposure to hypoxia. Data represents the average of seven independent experiments. (G) Representative images demonstrating demethylase activity of KDM4A in hypoxia. (H) KDM4A demethylase activity is retained following prolonged hypoxic exposure. RPE cells expressing 3xHA-WT-KDM4A were maintained in normoxia or hypoxia for 48 hours and H3K9 and H3K36 demethylation was assessed by immunofluorescence. The graph represents an average of two independent experiments with demethylase activity in hypoxia normalized to activity in normoxia. (I) Western blot depicting that JIB-04 treatment does not alter KDM4A protein levels upon hypoxia treatment. Lanes were spliced together from different regions of the same exposure of the same blot. (J) Cell cycle analysis following JIB-04 treatment demonstrating no difference in cell cycle phases. (K-N) Depletion of KDM5A and KDM6B does not rescue hypoxia-dependent copy gains. (K) Quantification of FISH for 1q12h and 8c in RPE cells maintained in normoxia or hypoxia with or without depletion of KDM5A or KDM6B. Data represents the average of two independent experiments performed with two independent siRNAs. (L) Western blot demonstrating siRNA depletion of KDM5A and CAIX induction in hypoxia. Lanes were spliced together from different regions of the same exposure of the same blot. (M,N) Quantification of siRNA-mediated depletion of KDM6B (M) and induction of CAIX (N) in normoxic or hypoxic RPE cells using qRT-PCR. Expression was normalized to β-actin and siCTRL in normoxia. (O) Western blot depicting that succinate does not alter KDM4A protein levels upon hypoxia treatment. Lanes were spliced together from different regions of the same exposure of the same blot. (P) Cell cycle analysis following succinate treatment demonstrating no difference in cell cycle phases. For all panels, error bars represent the S.E.M. and * indicates significant difference from control samples by two-tailed Student’s t-test (p<0.05).

**Fig. S5.** **Hypoxic tumor samples have copy gains of regions amplified in hypoxic cell culture.** (A) Analysis of cluster stability in TCGA breast cancer (BRCA) samples. The black hypoxic cluster remains stable at K=3 and higher. (B) Analysis of cluster stability in TCGA lung adenocarcinoma (LUAD) samples. The black hypoxic cluster remains stable at K=4 and higher. (C) Unbiased Hierarchical Clustering of BRCA samples by hypoxic gene signature. (D) Unbiased Hierarchical Clustering of LUAD samples by hypoxic gene signature. (E) TCGA Breast Cancer samples with a hypoxic gene signature have increased focal copy number gain. (F) TCGA Breast Cancer samples with a hypoxic gene signature have increased focal copy number loss. (G) TCGA lung adenocarcinoma samples with a hypoxic gene signature have increased focal copy number gain. (H) TCGA lung adenocarcinoma samples with a hypoxic gene signature have increased focal copy number loss. (I) Western blot depicting siRNA-directed depletion of KDM4A in normoxia and hypoxia.

**Supplemental Table S1. Spectral karyotyping results of cells grown in normoxia and hypoxia.**

**Supplemental Table S2. Amplified genes with expression changes in BRCA and LUAD primary tumor samples.**

**Supplemental Table S3. Hypoxia gene signature**

**References**

Black JC, Allen A, Van Rechem C, Forbes E, Longworth M, Tschop K, Rinehart C, Quiton J, Walsh R, Smallwood A et al. 2010. Conserved antagonism between JMJD2A/KDM4A and HP1gamma during cell cycle progression. *Mol Cell* **40**: 736-748.

Black JC, Manning AL, Van Rechem C, Kim J, Ladd B, Cho J, Pineda CM, Murphy N, Daniels DL, Montagna C et al. 2013. KDM4A lysine demethylase induces site-specific copy gain and rereplication of regions amplified in tumors. *Cell* **154**: 541-555.

Fu Y, Foden JA, Khayter C, Maeder ML, Reyon D, Joung JK, Sander JD. 2013. High-frequency off-target mutagenesis induced by CRISPR-Cas nucleases in human cells. *Nat Biotechnol* **31**: 822-826.

Fu Y, Reyon D, Joung JK. 2014. Targeted genome editing in human cells using CRISPR/Cas nucleases and truncated guide RNAs. *Methods Enzymol* **546**: 21-45.

Li B, Dewey CN. 2011. RSEM: accurate transcript quantification from RNA-Seq data with or without a reference genome. *BMC Bioinformatics* **12**: 323.

Manning AL, Longworth MS, Dyson NJ. 2010. Loss of pRB causes centromere dysfunction and chromosomal instability. *Genes Dev* **24**: 1364-1376.

Mermel CH, Schumacher SE, Hill B, Meyerson ML, Beroukhim R, Getz G. 2011. GISTIC2.0 facilitates sensitive and confident localization of the targets of focal somatic copy-number alteration in human cancers. *Genome Biol* **12**: R41.

Network TCGA. 2012. Comprehensive molecular portraits of human breast tumours. *Nature* **490**: 61-70.

Paw BH, Zon LI. 1999. Primary fibroblast cell culture. *Methods Cell Biol* **59**: 39-43.

Subramanian A, Tamayo P, Mootha VK, Mukherjee S, Ebert BL, Gillette MA, Paulovich A, Pomeroy SL, Golub TR, Lander ES et al. 2005. Gene set enrichment analysis: a knowledge-based approach for interpreting genome-wide expression profiles. *Proc Natl Acad Sci U S A* **102**: 15545-15550.

Van Rechem C, Black JC, Abbas T, Allen A, Rinehart CA, Yuan GC, Dutta A, Whetstine JR. 2011. The SKP1-Cul1-F-box and leucine-rich repeat protein 4 (SCF-FbxL4) ubiquitin ligase regulates lysine demethylase 4A (KDM4A)/Jumonji domain-containing 2A (JMJD2A) protein. *J Biol Chem* **286**: 30462-30470.

Van Rechem C, Black JC, Boukhali M, Aryee MJ, Graslund S, Haas W, Benes CH, Whetstine JR. 2015. Lysine Demethylase KDM4A Associates with Translation Machinery and Regulates Protein Synthesis. *Cancer Discov*.

Whetstine JR, Nottke A, Lan F, Huarte M, Smolikov S, Chen Z, Spooner E, Li E, Zhang G, Colaiacovo M et al. 2006. Reversal of histone lysine trimethylation by the JMJD2 family of histone demethylases. *Cell* **125**: 467-481.

Wilkerson MD, Hayes DN. 2010. ConsensusClusterPlus: a class discovery tool with confidence assessments and item tracking. *Bioinformatics* **26**: 1572-1573.

Winter SC, Buffa FM, Silva P, Miller C, Valentine HR, Turley H, Shah KA, Cox GJ, Corbridge RJ, Homer JJ et al. 2007. Relation of a hypoxia metagene derived from head and neck cancer to prognosis of multiple cancers. *Cancer Res* **67**: 3441-3449.
